# Promoter of *CaZF*, a Chickpea Gene That Positively Regulates Growth and Stress Tolerance, Is Activated by an AP2-Family Transcription Factor CAP2

**DOI:** 10.1371/journal.pone.0056737

**Published:** 2013-02-13

**Authors:** Deepti Jain, Debasis Chattopadhyay

**Affiliations:** National Institute of Plant Genome Research, Aruna Asaf Ali Marg, New Delhi, India; RIKEN Plant Science Center, Japan

## Abstract

Plants respond to different forms of stresses by inducing transcription of a common and distinct set of genes by concerted actions of a cascade of transcription regulators. We previously reported that a gene, *CaZF* encoding a C2H2-zinc finger family protein from chickpea (*Cicer arietinum*) imparted high salinity tolerance when expressed in tobacco plants. We report here that in addition to promoting tolerance against dehydration, salinity and high temperature, the *CaZF* overexpressing plants exhibited similar phenotype of growth and development like the plants overexpressing *CAP2*, encoding an AP2-family transcription factor from chickpea. To investigate any relationship between these two genes, we performed gene expression analysis in the overexpressing plants, promoter-reporter analysis and chromatin immunoprecipitation. A number of transcripts that exhibited enhanced accumulation upon expression of *CAP2* or *CaZF* in tobacco plants were found common. Transient expression of *CAP2* in chickpea leaves resulted in increased accumulation of *CaZF* transcript. Gel mobility shift and transient promoter-reporter assays suggested that CAP2 activates *CaZF* promoter by interacting with C-repeat elements (CRTs) in *CaZF* promoter. Chromatin immunoprecipitation (ChIP) assay demonstrated an *in vivo* interaction of CAP2 protein with *CaZF* promoter.

## Introduction

Environmental stresses such as changes in temperature, water and salt content in the soil are the major obstacles affecting plant growth and crop productivity. To cope with these stresses, plants undergo physiological and developmental adaptations that are manifested partly by altered gene expression. Changes in gene expression during stress responses have been extensively studied [1]–[3]. Transcriptional regulators are one of the important groups of proteins that contribute to the stress-adaptation process by regulating expression of genes that are important for changes in cellular metabolism in response to stress. Regulatory functions of a number of transcription factors, such as dehydration-responsive element binding proteins (DREB), basic helix-loop-helix (bHLH) proteins, MYB, MYC, WRKY and zinc-finger proteins have been described important for stress-adaptation [4]–[6].

Among several kinds of transcription factors involved in stress responses, the protein family with the Cysteine-2/Histidine-2-type (C2H2) zinc finger domains also called as the classical or TFIIIA-type finger is one of the best-characterized DNA-binding proteins found in eukaryotes [7], [8]. They were first identified in *Xenopus* oocytes [9]. These proteins have single or multiple canonical CX_2–4_CX_3_FX_5_LX_2_HX_3–5_H zinc finger motifs with two conserved cysteine and histidine residues. Plant C2H2 zinc finger proteins contain a non-variant QALGGH motif in each of the fingers which serves as a DNA binding motif. In plants the zinc finger proteins can have one to four fingers and the adjacent fingers are separated by a stretch of amino acids of variable lengths [10], [11]. The first plant C2H2 zinc finger protein ZPT2-1 (earlier named as EPF1) was identified from petunia for its ability to bind EP1S core element (TGACAGTGTCA) present in the promoter of its target gene *EPSPS* (*5-enolpyruvylshikimate-3-phosphate synthase*) [12]. Subsequently many other ZPT2-related zinc finger proteins having two zinc finger domains from different plant species were reported such as, WZF1 from wheat [13], SUPERMAN (SUP) [14] and AZF1 and AZF2 from *Arabidopsis* [15], Mszpt2-1 from *M. sativa* [16], SCOF-1 from soybean [17]. A number of them have been shown to play regulatory roles in plant growth and development, and also in stress response and defense activation pathways. STZ (ZAT10), an *Arabidopsis* TFIIIA-type zinc-finger protein reportedly provided drought-tolerance [18] and conferred tolerance to heat, salinity and osmotic stress when overexpressed in *Arabidopsis* [19]. *STZ* was also able to complement salt-sensitive phenotype of a yeast mutant [20]. Rice C2H2 zinc finger proteins ZFP179, ZFP182, ZFP245 and ZFP252 improved salt and drought tolerance in the overexpressing plants [21]–[24]. *Arabidopsis* proteins AZF1, AZF2, AZF3 and STZ were shown to repress transactivation abilities of DREB1A and AREB2 in an *Arabidopsis* protoplast transient analysis with chimeric promoter-reporter constructs [18]. These proteins possess a C-terminal DLNL/EAR (DLN box) sequence. This motif is also present in the proteins belonging to class II ethylene responsive factor (ERFII) family [25] and was shown to be essential for their function as transcription repressors.in plants [26]. Previously, we have shown that *CaZF* overexpression improved salt tolerance in transgenic tobacco [27]. Overexpression of different ZPT2-related zinc finger proteins have resulted in different phenotypes. While STZ, AZF1 and AZF2 were shown to function as transcription repressors; ectopic expression of STZ imparted drought-tolerance in contrast to the salt-sensitive phenotype of the plants overexpressing AZF1 and AZF2. Plants overexpressing any of these three genes displayed growth retardation [15]. However, ectopic expression of rice genes *ZFP252* or *ZFP179* did not exhibit any growth defect in rice [23], [24]. All these observations suggest that these proteins play diverse roles in different developmental and defense pathways.

The plant hormone abscisic acid (ABA) plays an important role in stress-responsive gene expression [28]. Previously, we identified CAP2, an AP2-family transcriptional regulator from chickpea. CAP2 functions as a C-Repeat binding factor (CBF) and binds to DRE/CRT (dehydration responsive element/C-repeat element) (CCGAC) that is often present in the promoter regions of abiotic-stress responsive genes. *CAP2* gene expression was induced by dehydration, high salinity and external ABA application. Ectopic expression of *CAP2* in tobacco resulted in improved tolerance to drought, salinity and heat and in addition, improved growth of the transgenic plants [29], [30]. In this study we reported that overexpression of *CaZF* in tobacco led to a similar phenotype of improved growth and stress-tolerance as in the case of *CAP2* overexpression. A number of tobacco transcripts that exhibited higher accumulation in the *CAP2*-expressing plants also showed higher abundance in the *CaZF*-expressing plants. Transient expression of *CAP2* in chickpea leaves enhanced *CaZF* gene expression. In a protoplast-mediated transient assay CAP2 induced expression of a reporter gene fused to the *CaZF* promoter sequence. Nucleotide substitution of C-repeat elements present in *CaZF* promoter suggested that CRTs are critical for CAP2-mediated activation of *CaZF* promoter. Chromatin immunoprecipitation followed by PCR-amplification indicated that CAP2 protein interacts with *CaZF* promoter *in vivo*. All together, our data demonstrated that CAP2 was able to tansactivate *CaZF* promoter *in planta* and indicated that CAP2 is a potential transactivator of *CaZF* gene.

## Materials and Methods

### Plant materials, Growth conditions and Stress treatments

Chickpea (*Cicer arietinum* L. cv. PUSABGD72 provided by Indian Agricultural Research Institute, New Delhi, India) was used in this study. 10-d-old seedlings were subjected to dehydration, salt, cold and hormonal treatments for given time periods (mentioned in the text) as discussed in [31]. Stress-treated seedlings were harvested, immediately flash-frozen in liquid nitrogen and stored at −80°C for later use. *CaZF*-overexpressing transgenic tobacco (*Nicotiana tabacum* var. *xanthii*) lines were constructed as described by [27]. T_3_ homozygous transgenic seeds were used for experiments. Vector transformed (Vec) and *CaZF-*overexpressing (*CaZF*OX) tobacco seeds were grown on ½ MS medium supplemented with 1.5% (w/v) sucrose, pH 5.8, and 0.8% (w/v) agar or in composite soil (agropeat∶vermiculite, 1∶1) at 25°C, 60% humidity and with a photoperiod of 16 h. Drought, mannitol and high temperature treatments were applied to the transgenic plants as described previously [29], [30]. Seedlings were harvested in liquid N_2_ and stored at −80°C for further analyses.

### Northern blot, qReal-Time PCR and Southern analysis

Total RNA was isolated from chickpea or tobacco seedlings using the TRI-reagent (Sigma-Aldrich, St. Louis, MO, USA) as per manufacturer's protocol. 20 µg of total RNA was electrophoresed on a 1.2% MOPS-formaldehyde denaturing agarose gel, blotted onto Nylon membrane (HyBond-N; Amersham Biosciences, Buckinghamshire, UK) and then hybridized with a random-prime labeled probe as described in [32]. Washed and dried membranes were then exposed in cassettes to storage phosphor screen for autoradiography. The autoradiographs were scanned on a PhosphorImager (Amersham Biosciences) and the relative intensities of the bands were quantitated using the ImageQuant program (Molecular Dynamics, Sunnyvale, CA). The values obtained were normalized by the background and then expressed as fold change with respect to control.

For semiquantative RT-PCR and qRT-PCR analysis total RNA was isolated from chickpea or tobacco seedlings using the RNeasy plant mini kit (Qiagen GmbH, Hilden, Germany). 2 µg of total RNA was used to prepare cDNA using High-Capacity cDNA Reverse Transcription kit (Applied Biosystems, Foster City, CA, USA). qRT-PCR reactions were performed in an optical 48-well plate in Applied Biosystems StepOne™ using PowerSYBR Green to monitor dsDNA synthesis. Chickpea and tobacco *Actin* primers were used as internal control. Sequences of all the primers used in this study are listed in Table S1.

The data presented are average of multiple biological replicates as mentioned in the results and figure legends. Error bars represent standard deviation. Statistical differences between the control and experimental samples were computed using Student's t-test with paired two-tailed distribution. The p-value cut-off was considered at p<0.005.

### Construction of subtracted cDNA library

Subtracted cDNA library was constructed between antibiotic selected Vec (driver) and *CaZF*OX (tester) tobacco by using CLONTECH PCR-Select cDNA subtraction kit (CLONTECH Laboratories, Palo Alto, CA) as described before [33]. Enriched DNA fragments from the forward subtracted library were directly cloned into T/A cloning vector (pGEM-T Easy Vector Systems, Promega, USA) and plasmids isolated from white colonies were further sequenced and annotated using BlastX with NCBI non-redundant protein sequence database (blast.ncbi.nlm.nih.gov).

### Analysis of transgenic tobacco plants for abiotic stress tolerance

T_3_ homozygous seeds of vector control (Vec) and transgenic *CaZF*OX tobacco lines were surface sterilized and grown essentially under culture room conditions as described previously [34]. For germination experiments, Vec and *CaZF*OX seeds were grown on ½ MS medium supplemented with 0.3 M mannitol as mentioned in the figures. Plates were photographed after 15 days. For germination under high temperature, the seeds were plated on ½ MS medium and kept at 39°C for 15 days. Seeds grown on ½ MS medium for the same period at control condition were taken as controls. For water-deficit stress of the soil grown plants, fresh weights of the stress-treated and recovered plants relative to those grown in control condition for the same period was calculated. All experiments were performed in triplicates and data in the form of mean of multiple experiments with standard deviation is presented. Proline content was determined in vector-control and *CaZF*OX transgenic plants under control and stress conditions by sulphosalicylic acid method [35].

For stress tolerance experiments of the soil-grown seedlings, two-week-old seedlings grown in composite soil at control condition were subjected to water deficit treatment. Stress tolerance was represented as fresh weight of the treated plants relative to those grown in control condition.

### Full length cloning and site directed mutagenesis of *CaZF* promoter

For *CaZF*-promoter (pro*CaZF*) cloning, 1.97 kb fragment upstream to the transcriptional start site of *CaZF* was amplified from a chickpea genomic DNA library constructed with Universal Genome Walker™ Kit (Clontech, USA) by following the manufacturer's protocol. *In silico* analysis of pro*CaZF* was performed by using PLACE signal scan search (http://www.dna.affrc.go.jp/PLACE/signalscan.html) [36] and PLANTCARE search (http://bioinformatics.psb.ugent.be/webtools/plantcare/html) [37] programs. *In vitro* site directed mutagenesis of the C-Repeat elements (CRTs) present at three different positions in pro*CaZF* was done by mutating “G” of the core motif (ACCGAC) to “T”.

For mutagenesis PCR various (CUM/F and CUM/R, M = M1, M2, M3) primer pair combinations (Table S1) were used. Mutagenesis PCR were done on double-stranded plasmid DNA using *Pfu* Turbo DNA polymerase (Stratagene, La Jolla, CA) by following parameters: 95°C for 30 sec; 16 cycles of 95°C for 30 sec, 58°C for 1 min, 72°C for 7 min. PCR reaction was digested with methylation-specific restriction enzyme *DpnI* at 37°C for 6 h for bacteria-derived template DNA degradation and the digested PCR product was directly transformed into *E. coli* DH5α cells. The mutation and the fidelity of the rest of the construct were confirmed by DNA sequencing.

### Gel mobility shift and promoter activation assay

For determining the *in vitro* binding activity of CAP2 with CRT present in pro*CaZF*, gel mobility shift assay (GMSA) was carried out as described by [27] with a radiolabeled probe containing tetramer of two CRTs (GACCGACCA) and its flanking sequences. The oligonucleotides used to amplify the CRT sequence are pCRT1 and pCRT2. Mutations in the probe sequences were done using the primers (pCRT3 and pCRT4) as mentioned in the figures. The recombinant GST-fused CAP2 protein was expressed in *E.coli* DH5α and purified from bacterial lysates with glutathione-Sepharose beads (Amersham Biosciences) as described in [29].

For promoter activation assay in yeast the pro*CaZF* was cloned in the *BamHI* restriction site of pYES2.1/V5-His/*LacZ* vector by using promoter specific primers, pyPro/F and pyPro/R to create the pYpro*CaZF:LacZ* construct. Site directed mutagenesis was done in the CRT3 site of the construct as described before and named as pYproM3*CaZF:LacZ*. *CAP2* ORF was amplified with primers pgCAP2/F and pgCAP2/R and cloned in *NdeI-EcoRI* restriction sites of pGADT7 (BD Biosciences Clontech) to create the pG*CAP2* construct. Both pG*CAP2* and pYpro*CaZF:LacZ* (or pYproM3*CaZF:LacZ*) were co-transformed in *his^−^leu^−^ Saccharomyces cerevisiae* strain AH109 by LiOAc method [27] and the transformants were selected on SD (-His/-Leu) medium. Transactivation property was assessed by *β-galactosidase* assay, done with three independent transformed colonies in triplicates by using ortho-nitrophenyl-β-D-galactoside (ONPG) as substrate.

### *In planta* promoter-reporter assay

For *in planta* GUS reporter and chromatin immunoprecipitation (ChIP) assay, pro*CaZF* was cloned in pBI101.1 before *β-glucuronidase* (pro*CaZF:GUS*, reporter plasmid) between *XbaI-BamHI* restriction sites and full length *CAP2* gene fused with two copies of *c-Myc* (2X*c-Myc*) epitope at the N-terminus was cloned between *NcoI-BglII* sites of pCAMBIA1302 (*CaMV35S:c-Myc-CAP2*, effector plasmid). Tobacco leaf-explants transformed by *Agrobacterium*-mediated method with pro*CaZF:GUS* and *CaMV35S:c-Myc-CAP2* individually or together were used for transient GUS expression analysis. The transgenic shoot-lets were selected on kanamycin and/or hygromycin supplemented media. Harvested shoot-lets were freezed in liquid N_2_ and crushed in extraction buffer for analyzing *GUS* activity. *NPT II* (Kanamycin resistance gene) expression, as assessed by qRT-PCR, was used for normalization. Average activity of fifteen shootlets of each source was presented.

### Protoplast isolation and transfection

Protoplasts were isolated from 5-d-old tobacco Bright Yellow-2 (BY-2) suspension cells, cultured and maintained in flasks at 28°C in dark, as described by [38] with few changes. Details of the protocol and solution recipes are mentioned in Methods S1. 10 µg DNA (control plasmids with or without pro*CaZF:GUS*/*CaMV35S:c-Myc-CAP2* plasmids) was mixed with 100 µl protoplasts (∼1×10^6^), followed by addition of 110 µl PEG (50%) solution and incubated at room temperature (RT) for 30 min. After incubation, 1 ml of W5 solution was added, centrifuged, re-suspended in 1 ml of incubation solution and incubated in dark at 28°C for 48 h. 5 µg of p*35S*:*EYFP1* plasmid (p*EYFP-1*; BD Biosciences Clontech, Palo Alto, CA, USA) [39] was included in each transfection experiment as a control for normalization of transfection efficiency.

### Protoplast extraction and quantitative GUS assays

Transfected protoplasts were collected by centrifugation at 1000 rpm, RT for 5 min, re-suspended in 500 µl of extraction buffer (100 mM sodium phosphate, pH 7.0, 0.5 mM PMSF) and disintegrated by vortexing. Total protein in the supernatants was measured by the Bradford method using bovine serum albumin (BSA) as the protein standard and GUS activity was measured using the *β-glucuronidase* substrate 4-methylumbelliferyl β-D-glucuronide (MUG) [40]. An average of GUS activity of three replicates was reported here. Two-step Student's t-tests were conducted between the samples containing reporter plasmid and reporter plasmid/effecter plasmid, and between the samples containing reporter plasmid/effecter plasmid and mutated reporter plasmid/effecter plasmid.

### Transformation by particle bombardment

*CaMV35S:c-Myc-CAP2* construct was introduced with control plasmid into leaf epidermal cells of potted chickpea (PUSABGD72) plant by particle bombardment using Helios gene gun (BioRad, www.bio-rad.com), according to the manufacturer's instructions. The leaf cells were bombarded with 1.0 µm gold particles at a helium pressure of 120 psi and a distance of 2 cm from the tissue. Plants were incubated at 25°C for 48 h to allow expression of transformed DNA. Leaf tissue bombarded with control plasmids only was taken as control. Control and two different experimental tissues were harvested after incubation period, flash frozen in liquid nitrogen and used for RNA isolation.

### Chromatin immunoprecipitation (ChIP)

Chromatin extraction and immunoprecipitation was performed essentially as described by [41] with slight modifications. Elaborate procedure with buffer compositions is described in Methods S1. Briefly, 1.5 g of tobacco shootlets harboring both pro*CaZF:GUS* and *CaMV35S:c-Myc-CAP2* constructs, grown under sterile conditions were harvested and fixed with 1% formaldehyde. Formaldehyde was quenched by adding glycine (final concentration, 0.3 M). Tissue was washed repeatedly with ice-cold Tris buffered saline (TBS). Fixed tissue was ground to a fine powder and resuspended in 1 ml lysis buffer I with protease inhibitors. The suspension was then sonicated and chromatin was pelleted at 13000 rpm, 4°C for 15 min. The supernatant was incubated overnight with preimmune serum or anti-*Myc* antibody (C-3956, Sigma-Aldrich, USA) and 40 µl Protein A-sepharose beads (Amersham Biosciences) at 4°C with gentle shaking. Beads were washed twice each with wash buffer, LNDET buffer and TE. The chromatin-immune complex was eluted and the DNA cross-linking was reversed by with NaCl. Protein was degraded by adding protease solution at 45°C for 2 h and DNA was precipitated with ethanol. Immunoprecipitated DNA was amplified with *CaZF*-promoter specific primers (Uchip/F and Uchip/R). In each PCR reaction, corresponding input was taken in parallel for PCR validation.

## Results

### *CaZF* expression analysis under different stress treatments

**Figure 1:**
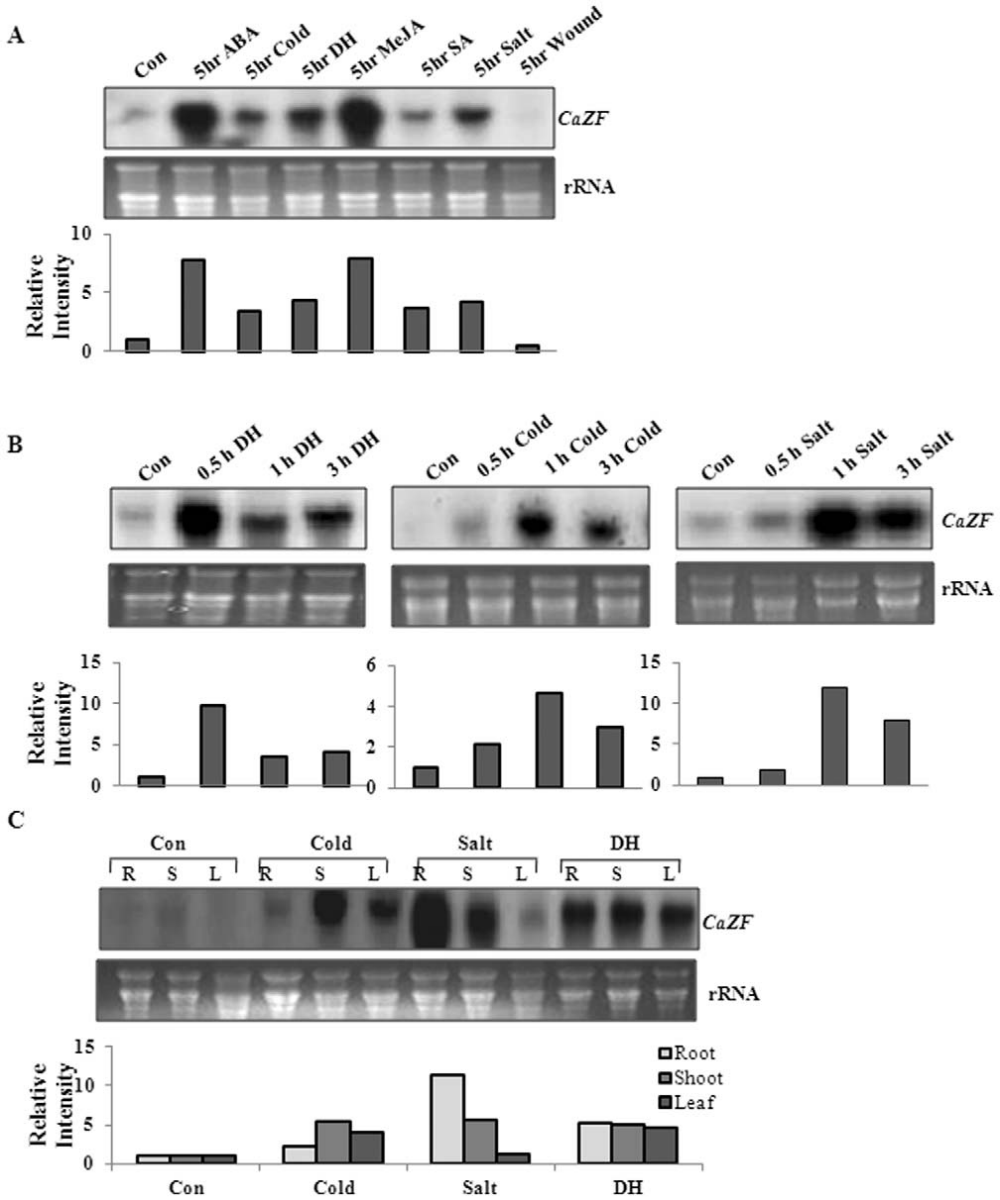
Expression analysis of *CaZF* in response to various stresses in chickpea. (A) Expression patterns of *CaZF* gene induced under different treatments. Total RNA was isolated from 10-d-old chickpea seedling treated with distilled water (control, Con), 100 µM abscisic acid (ABA), 4°C (Cold), dehydration (DH), 50 µM methyl-jasmonate (MeJA), 5 mM salicylic acid (SA), 250 mM NaCl (Salt) and wound for 5 h. (B) Time-course of accumulation of *CaZF* mRNA under dehydration, cold and salt treatments for 0.5 h, 1 h and 3 h. (C) Organ-specific expression of *CaZF* in root (R), shoot (S) and leaf (L) after cold, salt and dehydration treatments for 3 h. 20 µg of total RNA was electrophoresed on formaldehyde denaturing gel, blotted onto nylon membrane and hybridized with ^32^P-radiolabeled *CaZF* cDNA. The lower panel in each figure shows ethidium bromide-stained ribosomal RNAs as loading controls. The relative intensities of the bands were quantitated by densitometry in PhosphorImager scanner and are presented in the form of fold changes below each blot.

We have reported earlier the cloning of *CaZF* from chickpea [27]. Briefly, CaZF is a Cys2-His2 zinc finger protein of 280 amino acids and is nuclear localized. The expression of *CaZF* in 10-d-old chickpea seedling was monitored by northern blot analysis under different stress conditions and hormonal treatments. Northern blot analysis with a *CaZF* specific probe showed a basal level of expression under control condition. Increased steady-state accumulation of *CaZF* transcript was observed after 5 hour (h) of treatment with low temperature, dehydration (DH), salt, abscisic acid (ABA), methyl-jasmonate (MeJA) and salicylic acid (SA), except wounding (Figure 1A). The time-dependent expression kinetics of *CaZF* under dehydration, high salinity and low temperature showed that the transcript quickly accumulated at a ten-fold higher level within 0.5 h under dehydration and then reduced at 3.5–4-fold higher up to 3 h of treatment. Similarly, two-fold high transcript accumulation was observed within 0.5 h of cold treatment, and then further reached to five-fold high after 1 h followed by a reduction to three-fold after 3 h. While under salt stress, two-fold increase in transcript accumulation was observed within 0.5 h and further accumulated up to 12-fold after 1 h and decreased to 8-fold at 3 h (Figure 1B). Organ-specific expression of *CaZF* was observed in root, stem and leaves in normal and stressed conditions. Expression was strongly induced in all the tissues under dehydration stress, specifically in root and stem under salt stress and in stem under cold treatment (Figure 1C).

### Phenotypic features of transgenic tobacco plants constitutively expressing *CaZF*

**Figure 2:**
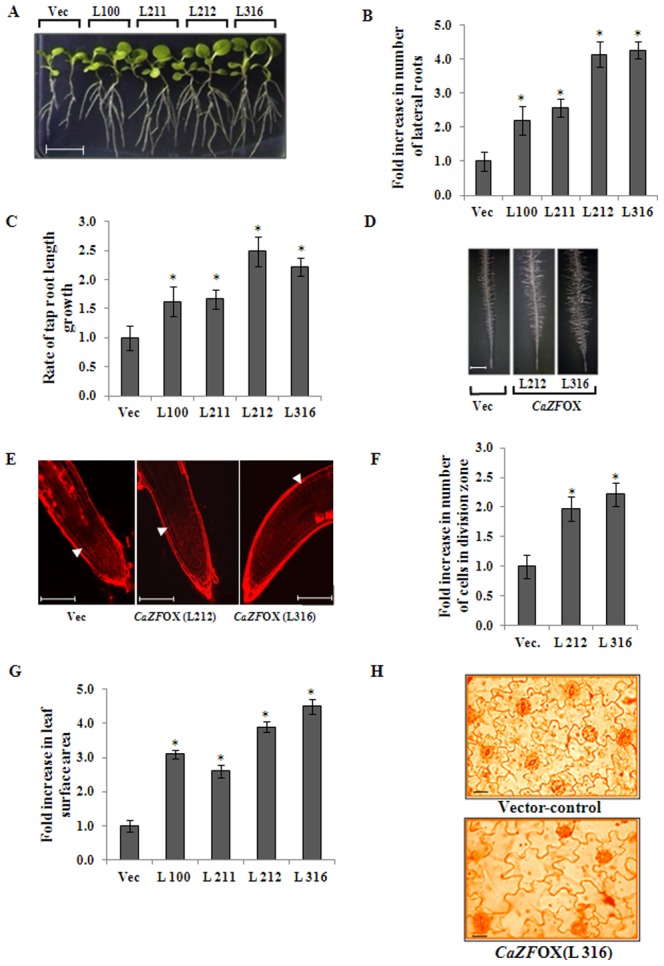
Phenotype analyses of *CaZF* overexpressing (*CaZF*OX) transgenic tobacco plants. (A) Tobacco seedlings of four independent T_3_ homozygous lines harboring *CaZF* (*CaZF*OX, L100, L211, L212, L316) or empty vector (Vec) were grown vertically under control conditions (½ MS, 25°C) for 15 days. Scale bar is 1 cm. (B) Comparison of number of lateral roots, and (C) fold increase in rate of taproot length growth of vector-control and *CaZF*OX seedlings. The means of three measurements of thirty seedlings of each line are shown. (D) Comparison of root hair of 6-day-old vector-control and *CaZF*OX (L212 and L316) tobacco seedlings. Scale bar is 1 mm. (E) Confocal imaging of root apices of 6 d-old vector-control and *CaZF*OX tobacco seedlings stained with fluorescent dye Propidium iodide (PI) at equal magnification. Arrow marks the start of elongation zone. Scale bar is 100 µm. (F) Graphical representation of fold increase in number of dividing cells in the meristematic zone of roots of 6-day-old *CaZF*OX plants is presented in the right panel. (G) Comparison of surface area of the third (from the bottom) leaves of 15-day-old vector-control and *CaZF*OX plants. (H) Epidermal peels from the ventral surface of the middle lamina of the vector control and transgenic (*CaZF*OXL316) leaves showing cell size. Scale bar is 10 µm. The error bars indicate the standard deviation (SD). * indicates significant differences in comparison with the vector-control (Vec) at p<0.005.

In our previous study, we reported construction of transgenic tobacco plants overexpressing *CaZF* (*CaZF*OX lines) under *CaMV35S* promoter [27]. Two single copy insertion homozygous lines (from T_3_ homozygous seeds) each with relatively high (*CaZF*OX lines L212 and L316) and low expressing (*CaZF*OX lines L100 and L211) *CaZF* were selected for further experiments. No differences in germination period and frequency were observed between the transgenic and the control (Vec) seeds on control medium (half strength MS medium, ½ MS, 25°C). However, 15-day-old vertically grown seedlings showed a number of morphological differences in comparison to the vector-control plants. Fifteen-day-old transgenic seedlings showed enhanced growth and 2–4 fold increase in the number of lateral roots in comparison to the control seedlings of same stage (Figure 2A, B). Tap roots of *CaZF*OX lines exhibited more than two fold increase in average length for L212 and L316, and about 1.5 fold increase for L100 and L211 (measured over the period of consecutive three days) compared to the control seedlings (Figure 2C). *CaZF*OX lines also showed longer and robust root hair (Figure 2D). Longer root phenotype in *CaZF-*overexpressing lines was investigated for altered cellular organization at the root tip by confocal imaging (Figure 2E). *CaZF*OX root tips showed no gross deformation in the cellular arrangement. However, roots of *CaZF*OX lines showed a relative increase of approximately two-fold in the number of dividing cells of the meristematic region (Figure 2F). Increased cell number was evident in the epidermal, cortical and stellar layers and that caused increase in cell layers in *CaZF*OX roots indicating enhanced cell division. Subsequently to the enhanced cell division, the zone of cell division is also longer in these seedlings.

Enhanced growth phenotype of 15-day-old transgenic seedlings was clearly depicted by larger leaf lamina. Average surface area of the third (from the bottom) leaves of 10 plants from each transgenic line was measured and found to be approximately 2.5–4.5 fold higher in *CaZF*OX plants in comparison to the control samples (Figure 2G). To explore the reason behind the increase in the leaf lamina in the transgenic plants, the ventral epidermal peel from both sides of mid-rib in the leaf base, middle lamina and leaf tip of the vector control and *CaZF*OX lines were compared for cell size. Six samples from each leaf and leaves from two plants of each line were investigated. Leaf cell size of the *CaZF*OX plants was found to be larger than that of the control leaves (Figure 2H).

### *CaZF* overexpression enhances germination efficiency of the seeds under abiotic stresses

**Figure 3:**
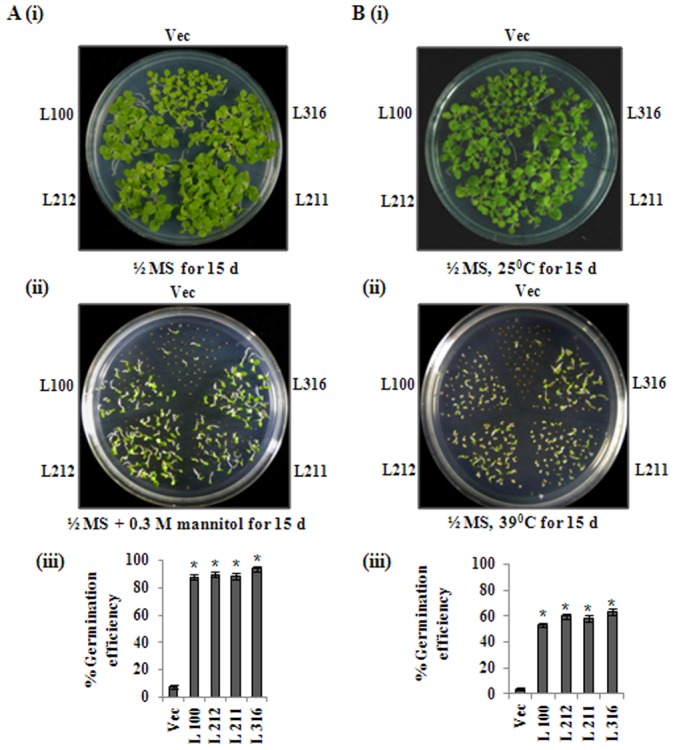
Germination efficiency of *CaZF*OX tobacco lines in mannitol and under high temperature. Seeds of *CaZF*OX transgenic lines (L100, L212, L211 and L316) and vector-control (Vec) plant were sown on ½ MS-agar [A (i), B (i)] or ½ MS-agar supplemented with 0.3 M mannitol [A (ii)] and incubated for 15 days at 25°C for germination. Seeds from the same plants were plated on ½ MS-agar and incubated at 39°C for 15 days for high temperature treatment [B (ii)]. Graphical representation of percent germination of seeds after 15 days on incubation from three independent experiments is presented below [A (iii), B (iii)]. The error bars indicate the standard deviation (SD). * indicates significant differences in comparison with the vector-control (Vec) at p<0.005.

We previously showed that seeds of *CaZF*OX plants displayed enhanced germination efficiency under salt stress (27). Here, we evaluated the effects of osmotic and high temperature stresses on seed germination of *CaZF*OX plants. T3 homozygous seeds of vector-control and *CaZF*OX lines were allowed to germinate on **½** MS-agar supplemented with 0.3 M mannitol at 25°C for osmotic stress treatment or on **½** MS-agar at 39°C for high temperature treatment [Figure 3A(ii), B(ii)]. Germination of seeds was delayed under stress as compared to that in the control condition. The germination efficiency of *CaZF*OX lines was above 85% in comparison to about 8% for the vector-control seeds after 15 days of sowing on mannitol-supplemented medium [Figure 3A (iii)]. Under higher temperature, only 4% vector-control seeds showed germination after 15 days, while *CaZF*OX seeds showed germination efficiency above 55% [Figure 3B (iii)]. Seeds of vector-control and *CaZF*OX plants did not show any difference in germination period and frequency in control media [Figure 3A (i), B (i)].

### Stress tolerance of soil-grown *CaZF*OX transgenic plants

**Figure 4:**
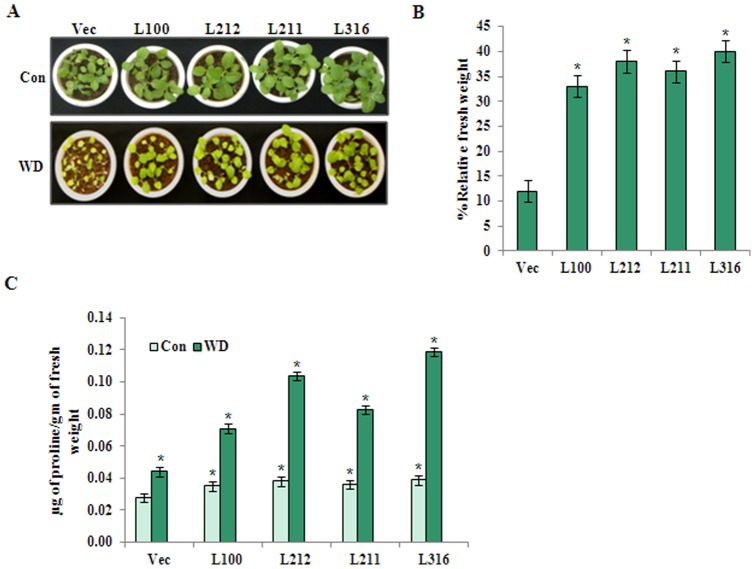
Abiotic stress response of soil grown *CaZF*OX tobacco plants. (A) Fifteen-day-old vector-control (Vec) and *CaZF*OX seedlings grown in control condition were not watered for two weeks and then watered for one week for recovery (WD). Plants grown at control condition for the same period is shown in the upper panel (Con). (B) Comparison of relative fresh weights of the plants treated with water deficit stress. (C) Comparison of proline content between 15-day-old vector-control and *CaZF*OX transgenic lines under control condition and water deficit stress (WD) treatment for two-weeks. The error bars indicate the standard deviation (SD). * indicates significant differences in comparison with the vector-control (Vec) at p<0.005.

We monitored the stress-tolerance of the soil-grown transgenic lines under water-deficit (WD) condition. Percent fresh weight under stress conditions relative to that grown in control condition was presented to compare the stress-tolerance of the control and transgenic plants. Fifteen-day-old vector control and *CaZF*OX plants grown in control condition were not irrigated for 2 weeks for water deficit stress. For recovery, pots were watered for a week. *CaZF*OX transgenic seedlings recovered more than the vector control plants, which appeared to be mostly withered (Figure 4A). As shown in Figure 4B, the *CaZF*OX seedlings retained 32–40% of relative fresh weight, whereas vector-control seedlings retained only 10–15% fresh weight compared with the unstressed seedlings.

To investigate the physiological basis for the improved stress tolerance of transgenic tobacco, proline content was measured in 15-day-old *CaZF*OX and vector-control plants under control and stressed conditions. After water-deficit stress, *CaZF*OX plants accumulated approximately 1.5 to 3-fold higher amount of proline than the vector-control plants. Under control condition,vector-control and *CaZF*OX plants accumulated similar amount of proline, indicating that other stress regulated factors are required along with *CaZF* to regulate the accumulation of proline under stress (Figure 4C).

### Analysis of altered gene expression due to *CaZF* overexpression in transgenic plants

Physiological and biochemical experiments suggested that *CaZF* overexpression might have altered expression of some genes related to abiotic stress signaling and helped in the cellular survival and recovery. To gain an insight into alteration of gene expression, specifically to identify the up regulated genes in the *CaZF*-transgenic plants, a subtractive cDNA library was constructed with the mRNAs isolated from the 2-week-old seedlings of vector control (driver) and *CaZF*OX (tester) lines grown in control condition. After screening out the redundant sequences, 222 high-quality unique EST sequences were generated, annotated and classified according to their putative functions (Table S2). Many of the well-known abiotic stress-responsive genes such as HSFs, *CBF/DREB*, catalase, *CIPK16*, Ca^2+^-binding proteins and MAPK1 were identified, indicating their likely contribution towards the stress tolerance of transgenics. For validation of enhanced expression of the ESTs, expression of 6 ESTs (MRP-like ABC transporter, Auxin efflux carrier family protein-like protein, Heat-shock factor, Lipid transfer protein, *CIPK16* and Elongation-factor 1α) was verified by qRT-PCR (Figure S1A–F).

### CAP2 activates *CaZF* promoter

*CAP2* overexpression in tobacco plants caused drastic increase in leaf cell size, leaf surface area and in the number of lateral roots. Further, the *CAP2*-expressing plants demonstrated more tolerance to water-deficit and salt stress [29]. *CaZF* overexpression resulted in similar developmental and stress tolerance phenotypes as that of *CAP2*-overexpressing plants. In addition, a number of ESTs identified in *CaZF*OX subtracted cDNA library (Dehydrin, Heat shock factor, Heat-shock protein 70, Heat-shock protein 90, Secretory peroxidase, Glyceraldehyde-3-phosphate dehydrogenase, Myo-inositol-1-phosphate synthase and H^+^-transporting ATPase) were found to be similar to those in *CAP2*OX subtracted cDNA library [30]. Expression of abiotic stress marker genes like *NtERD10B* and *NtERD10C* was similarly enhanced in the *CaZF* and *CAP2* transgenic seedlings under unstressed conditions (Figure S1G–H) [this report and [29]. Similarity of the phenotypes of *CAP2*OX and *CaZF*OX transgenic plants and their gene expression prompted us to investigate a relationship between these two genes.

**Figure 5:**
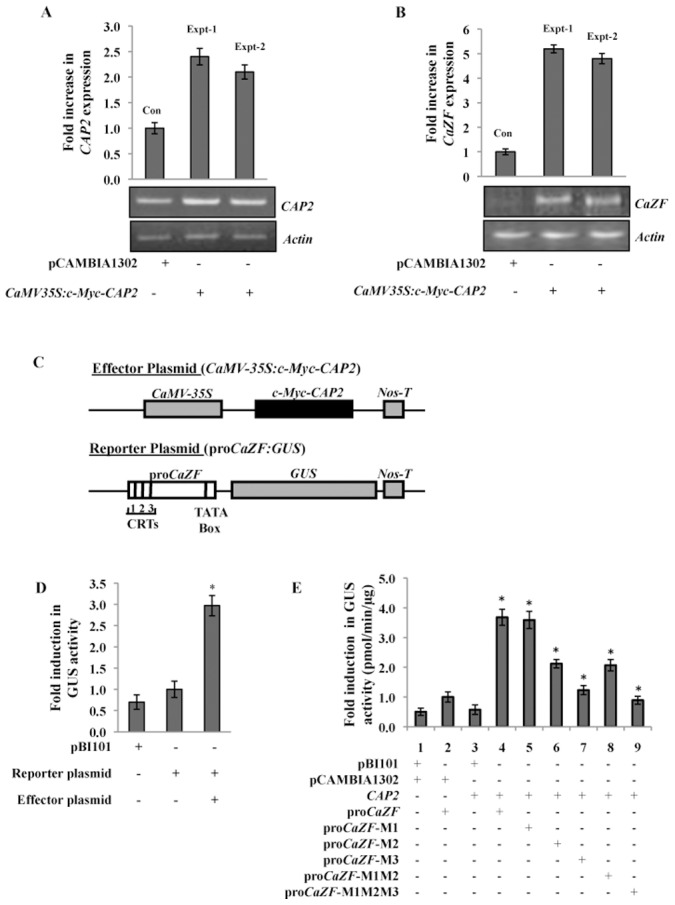
Activation of *CaZF*-promoter by CAP2 in plant cell. (A, B) Enhancement of *CaZF* expression by transient overexpression of *CAP2* in chickpea. Young leaves of 10-day-old chickpea seedlings were transformed with *CaMV35S:c-Myc-CAP2* by particle bombardment. Chickpea leaves were harvested after 48 h of incubation. Leaves transformed with empty vector (pCAMBIA1302) were taken as control (Con). 2 µg of total RNA was reverse transcribed for cDNA preparation. *CAP2* (A) and *CaZF* (B) expression was analyzed in the control and experimental tissues by semi-quantitative RT-PCR (27 cycles) and qReal-Time PCR. The expression level of *Actin* gene was taken as an internal control. Results from two biological replicates (Expt-1, Expt-2) are shown. (C) Schematic diagram of the effector and reporter constructs used in the co-transfection experiments. Full-length *CAP2* cDNA was fused with 2X*c-Myc* at N-terminus and cloned under *CaMV-35S* promoter in pCAMBIA1302 to construct effector plasmid. pro*CaZF* with three CRTs was fused with *GUS* gene in pBI101 to construct reporter plasmid. (D) Both the effector and reporter plasmids as mentioned in the figure were co-introduced in to tobacco leaf explants by *Agrobacterium*-mediated transformation and antibiotic-selected shootlets were used for the GUS assay. Expression of kanamycin resistance gene (NPT II) as assessed by qRT-PCR was used for normalization of results in the transformed shoot-lets. (E) The effector and reporter constructs were co-introduced into tobacco BY2 protoplasts as mentioned in the table. *CAP2* stands for the effector plasmid and pro-*CaZF* stands for the reporter plasmid. pro-*CaZF* (M1–M3) stands for the reporter plasmids with mutations in CRT1-CRT3 in pro-*CaZF*. GUS activity was measured fluorometrically after 48 h of transformation. The empty vectors without *CAP2* (pCAMBIA1302) or pro*CaZF* (pBI101) were used as controls. Transfection efficiency of the *CaMV-35S-EYFP1* plasmid included in protoplast experiment was used for normalization. The error bars indicate the standard deviation (SD). * indicates significant differences in comparison to the controls at p<0.005.

To investigate whether *CAP2* expression enhances *CaZF* expression in chickpea, we transiently expressed *CAP2* in chickpea leaves by introducing *CAP2* expression construct (*CaMV35S:c-Myc-CAP2*) in chickpea leaves by particle bombardment resulting in increase in *CAP2* expression (Figure 5A). Subsequently, expression of *CaZF* was increased by about five folds as determined by semi-quantitative RT-PCR and qRT-PCR (Figure 5B). To further elucidate the effect of *CAP2* expression on *CaZF* expression, 1970 bp of *CaZF* promoter (pro*CaZF*) region was cloned by genome walking. Bioinformatics analysis of pro*CaZF* showed presence of three authentic C-repeat elements (CRT: CCGAC) at the positions −1913 (CRT1), −1713 (CRT2) and −1337 (CRT3) along with putative abscisic acid responsive elements, auxin-responsive elements, MYC and MYB binding sites upstream to the transcription start site (Figure S2). 1.97 kb *CaZF* promoter was fused to *β-glucuronidase* (*GUS*) reporter gene to make a reporter construct (pro*CaZF*:*GUS*) (Figure 5C). This reporter and the effector constructs (*CaMV35S:c-Myc-CAP2*) were introduced individually or together in tobacco leaf explants by *Agrobacterium*-mediated transformation. GUS activity in the antibiotic selected shootlets was found to be enhanced about 3-fold in presence of CAP2 (Figure 5D). Ability of CAP2 to activate *CaZF* promoter in *Saccharomyces cerevisiae* was also analyzed. Expression of the reporter gene was increased by more than 80-fold in presence of CAP2 (Figure S3), demonstrating that CAP2 was able to activate pro*CaZF*. In order to dissect the roles of three CRTs in *CaZF* promoter in transcriptional regulation, these CRTs were mutated individually or in combination by replacing ‘G’ with ‘T’ (CCGAC/CCTAC). Effect of these substitutions in transcription activation of the reporter construct was tested in the tobacco BY2 protoplast transient assay system by co-introduction of the mutated reporter constructs along with the effector construct (*CaMV35S:c-Myc-CAP2*). Figure 5E shows that presence of CAP2 led to almost 4-fold enhancement of GUS activity. Mutation (M1) in CRT1, which is the farthest (−1913) from the transcription start site, did not alter the GUS activity significantly, however, mutation in CRT2 (M2, −1713) and CRT3 (M3, −1337) significantly reduced the GUS activity; indicating critical role of these CRTs in CAP2 mediated *CaZF* promoter activation. Mutations in combination also suggested that CRT2 and CRT3 are the most important for CAP2-mediated activation of *CaZF* promoter.

### CAP2 binds to *CaZF* promoter

**Figure 6:**
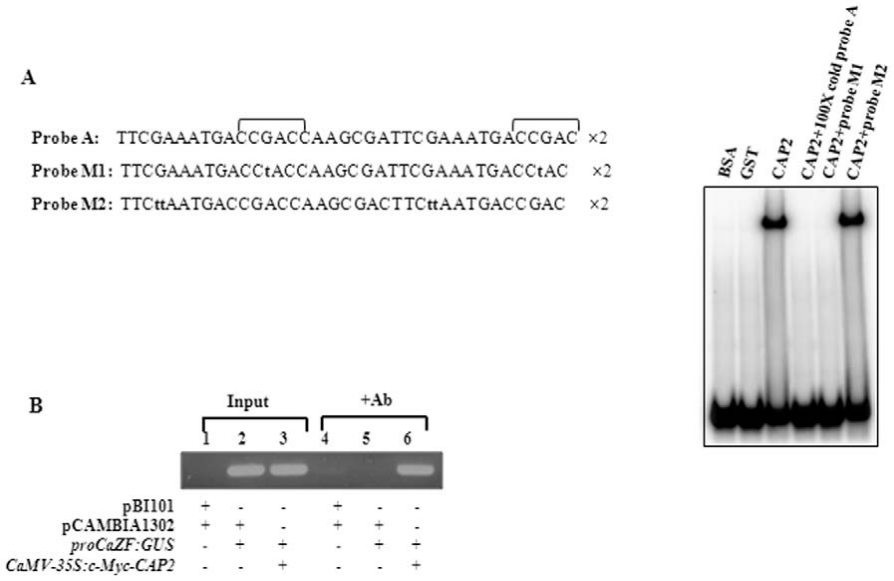
*CaZF* promoter is a target of CAP2. (A) The DNA binding ability of CAP2 to CRT in *CaZF* promoter was analyzed by gel mobility-shift assay (GMSA). Dimers of the sequences shown in the left panel were used as probes. CRT nucleotides are shown in bracket and the mutations (M1 and M2) created within the probe by replacing bases are shown in lower case. GMSA was performed with purified GST-CAP2 fusion protein expressed in bacteria using cold or ^32^P-labeled probe. (B) ChIP assay indicating CAP2 interacts with *CaZF* promoter *in vivo*. Tobacco leaf explants were transformed with pro*CaZF:GUS* and *CaMV-35S:c-Myc-CAP2* constructs along with control plasmids. Antibiotic-selected shootlets were harvested and fixed with 1% formaldehyde. The DNA-protein complex was immunoprecipitated by anti*-c-Myc* antibodies. Quantitative PCR was performed using pro*CaZF* specific primers flanking the CRT3 region. Lanes 1–3, Input (total DNA-protein complex); lanes 4–6, DNA-protein complex immunoprecipitated with anti-*c-Myc* antibody. Empty plasmids without pro*CaZF* (pBI101) or *c-Myc-CAP2* (pCAMBIA1302) were used as controls.

We reported earlier the sequence specific interaction of CAP2 protein with CRT [29]. In order to analyze whether CAP2 protein can directly bind to the CRT present in *CaZF* promoter, we performed GMSA with different DNA probes containing repeat sequence of CRT3 and it's flanking bases and purified *E. coli-*expressed CAP2 protein fused in-frame to glutathione-S-transferase (GST). Figure 6A clearly shows that GST-CAP2 protein could specifically bind to the radiolabeled probe as the binding was competed out by 100× cold probe. The *in vitro* binding of CAP2 protein to the probe was sequence-specific, as replacement of ‘G’ with ‘T’ within the CRT (CCGAC/CCTAC) abolished the binding while change in the flanking sequences did not affect the binding.

Finally, to determine whether the CAP2 protein interacts with *CaZF* promoter within the plant cell we performed chromatin immunoprecipitation (ChIP) assay. The *CaMV35S:c-Myc-CAP2* fusion construct was co-introduced along with pro*CaZF:GUS* construct into tobacco explants by *Agrobacterium*-mediated transformation. Antibiotic-selected transformed shoot lets harboring both pro*CaZF:GUS* and/or *CaMV35S:c-Myc-CAP2* were harvested and fixed with 1% formaldehyde in fixation buffer. The DNA-protein complex was immunoprecipitated by anti-*c-Myc* antibody. The DNA fragments that co-immunoprecipitated with anti-*c-Myc* antibody were identified by PCR amplification using the primers containing the flanking bases of CRT3 of the *CaZF* promoter (Figure 6B). Altogether, the results revealed a strong interaction of CAP2 with the *CaZF* promoter within the plant cell.

## Discussion

We presented this study as a continuation of our previous study [27] where we reported that *CaZF* can provide salt-tolerance when expressed in tobacco plants. CaZF belongs to zinc finger family of proteins and contains two typical Cys2/His2 zinc finger domains with a DLN-box/EAR-motif at its C-terminus. A number of similar plant Cys2/His2 zinc finger proteins, such as STZ/ZAT10, AZF1, AZF2 were shown to repress transactivation properties of the well-known transcription activators like DREB1A and AREB2 in transient assays [18]. Like CaZF, Cys2/His2 zinc finger proteins ZFP179 from rice [24] and ThZF1 from salt cress [42], showed transactivation property in yeast system. Repressor activity of C2H2 zinc finger proteins having EAR motif is mediated through interaction with TOPLESS and its related proteins in plants [26]. Therefore, possibility of transactivation of reporter genes by CaZF in yeast system due to lack of EAR motif-TOPLESS pathway in yeast cannot be ruled out.

Inducible expression of *AZF1* and *AZF2* resulted in severe salt-sensitivity, however, constitutive expression of *STZ/ZAT10* promoted drought-tolerance and; *CaZF* and *ZFP179* promoted salt-tolerance when overexpressed in plants (this report and [24]). Therefore, it appears that *in planta* functions of this family of proteins cannot be generalized just on the basis of structural homology. Though these proteins were shown to bind the same sequence motif (AG/CT repeat) within an EP2 sequence in *in vitro* experiments, optimal binding efficiency of these proteins to their target sequences within a plant cell and its effect on expression of the target genes or requirement of other auxiliary proteins seems to play a major role in determining their functions in plant. To elaborate, overexpression of *STZ*, *ZFP179* and *CaZF* enhanced expression of the oxidative stress response genes in their respective systems e.g. overexpression of *STZ* enhanced ascorbate peroxidase 2, and Fe-superoxide dismutase 1 [18]; overexpression of *ZFP179* enhanced peroxidase activity in the transgenic rice [24] and overexpression of *CaZF* enhanced expression of catalase and secretory peroxidase genes in tobacco. However, transcript levels of similar ROS-responsive genes were not altered in *AZF1* and *AZF2* overexpressing plants. Inducible expression of *AZF1* and *AZF2* repressed the expression of *DREB1A* [15], while the overexpression of *ZFP179* enhanced expression of *DREB2A* [24] and overexpression of *CaZF* enhanced expression of *DREB3* and *DREB4*. Enhancement or reduction in expression of the DREB-family genes in these overexpressing plants does not necessarily indicate that those DREB-family genes are under transcriptional control of these zinc finger proteins. Expression of all the four zinc finger protein-encoding genes mentioned here is highly enhanced by external application of ABA, whereas, *DREB1A* and *DREB2A* genes are not ABA-responsive. Alteration in expression of these DREB-family genes in these overexpressing lines might be direct or, due to any feed-back response.

Many plants accumulate cellular osmolytes like proline for adjusting the intracellular osmotic potential under abiotic stress [43]. Thus, proline accumulation was measured to explain one of the possible reasons for enhanced abiotic stress tolerance of *CaZF*-overexpressing plants. Although *CaZF*OX plants exhibited higher proline content in stressed conditions, we did not detect any clone encoding proline synthetase gene (*P5CS*) in the subtracted cDNA library. The reason might be similar to that in the *ZFP179*-overexpressing plants, where there was no enhancement in *OsP5CS* gene expression in unstressed condition, however, the gene expression was much higher in the overexpressing plants under stressed condition suggesting that other stress-dependent factors are also required for enhancement in expression of some of the stress-responsive genes. Enhanced growth phenotype of the *CaZF*-overexpressing plants can be attributed to elevated expression of ESTs encoding auxin-efflux carrier protein, AUX/IAA and ABC-transporters (Figure S1) and, thereby, increased auxin transport (Figure S4 and Methods S1). For instance, the retarded growth phenotype of *AZF2*-overexpressing plants can be attributed to the down regulation of expression of some of the auxin-responsive genes [15].

*CaZF* promoter, like the promoters of *AZF* and *ZFP179* possesses multiple C-repeat and ABA-responsive elements. Apart from that it also contains five auxin-responsive elements (Figure S2). Regulation of these promoters by AREBs and ARFs is subject to experimentation.

In summary, we have presented a detail study of the effect of *CaZF*-overexpression in plant. *CaZF*-expression promoted tolerance of the transgenic plants against dehydration and high temperature along with an overall growth in the aerial and subaerial parts of the plants. We have shown that transient expression of *CAP2*, a chickpea gene for CBF/DREB-like transcription factor, caused enhanced accumulation of *CaZF* transcript in chickpea leaves. CAP2 protein was able to bind to C-Repeat/dehydration-responsive element present in the *CaZF* promoter and activate transcription as determined by GMSA and protoplast transient transactivation assay respectively. Finally, chromatin immunoprecipitation (ChIP) suggested that CAP2 has ability to bind *CaZF* promoter in plant cell. Our results indicated that CAP2 is a potential transactivator of *CaZF*.

## Supporting Information

Table S1 **Oligonucleotide sequences used in this study.**
(DOC)

Table S2 **Functional categorization of ESTs generated by subtracted cDNA library between** ***CaZF*** **OX and vector control.**
(DOC)

Figure S1 **Quantitative RT-PCR of selected genes. (A–F) mRNA abundance of some of the library genes was determined by qRT-PCR.** GO308103, MRP-like ABC transporter; GO308100, Auxin efflux carrier family protein-like protein; GO308183, Heat-shock factor; GO308200, Lipid transfer protein; GO308212, CIPK16 and GO308225, Elongation-factor 1α. (G–H) Expression of abiotic stress marker genes *NtERD10B* (AB049336) and *NtERD10C* (AB049337) in vector control and *CaZF*-overexpressing transgenic tobacco lines was analyzed by quantitative Real-time PCR in normal growth condition. Tobacco *Actin* is used as an internal control for normalization. Data shown here is a mean of three independent experiments. The error bars indicate the standard deviation (SD). * indicates significant differences in comparison with the vector-control (Vec) at p<0.005.
(TIF)

Figure S2 ***In-silico*** **sequence analysis of** ***CaZF*** **-promoter (pro** ***CaZF*** **).** *CaZF* promoter region was analyzed by PLACE and PlantCare softwares. Some of the elements worth to mention are highlighted. Abscisic acid responsive element as bold *italics*, C-repeat elements (CRTs) as bold underline, auxin-responsive elements [ARFAT site (TGTCTC), CATATGGMSAUR site (CATATG), NTBBF1ARROLB site (ACTTTA), DOFCOREZM site (AAAG) and TGA Element (AACGAC)] as lowercase underline, MYC consensus as underline and MYB consensus as bold lowercase.
(TIF)

Figure S3 **CAP2 transactivate pro** ***CaZF*** **in yeast.** Activation of reporter gene *LacZ* under *CaZF* promoter by CAP2 in *Saccharomyces cerevisiae*. Activation of reporter gene *LacZ* under *CaZF* promoter by CAP2 in *Saccharomyces cerevisiae*. A, Yeast strain PJ69-4A co-transformed with the constructs indicated in the left plate were grown on SD (-His, -Ura) or in SD complete medium. B, Schematic representation of effector and reporter constructs. C, Interaction of pro*CaZF* with CAP2 in yeast. Yeast strain BCY213 was co-transformed with the constructs as indicated in model plate. *Trans*-activation of pro*CaZF* by CAP2 is shown by *β-galactosidase* assay of the transformants. Activity is presented as fold increase in activity. Assay was done by taking three independent transformed colonies in triplets. The error bars indicate the standard deviation (SD). * indicates significant differences in comparison with the pYpro*CaZF:LacZ* (Lane 2) at p<0.005.
(TIF)

Figure S4 **Basipetal-auxin transport assay in the roots of the vector-control and** ***CaZF*** **OX plants.** Comparison of basipetal root auxin transport in 8-d-old vector control (Vec) and *CaZF*OX (L100, L212, L211 and L316) seedlings in root segments above the site of auxin application at the root tip. An average of auxin transport in root segments was calculated and data presented was fold increase in average auxin transport, relative to vector control tobacco. Results from three independent experiments are presented. The error bars indicate the standard deviation (SD). * indicates significant differences in comparison with the vector-control (Vec) at p<0.05.
(TIF)

Methods S1 **Protoplast isolation and co-transfection analysis, Chromatin immunoprecipitation (ChIP) and Auxin transport assay.**
(DOC)
