# Supplementary material for: Promoter of CaZF, a Chickpea Gene That Positively Regulates Growth and Stress Tolerance, Is Activated by an AP2-Family Transcription Factor CAP2
Source: PLoS One. 2013 Feb 13;8(2):e56737. doi: 10.1371/journal.pone.0056737 (PMC3572041; doi:10.1371/journal.pone.0056737)
Supplement: Table S1 — Oligonucleotide sequences used in this study. (DOC) [file pone.0056737.s001.doc]

**Table S1.** Oligonucleotide sequences used in this study

| **S. No.** | **Name of the Oligo** | **Sequence (5’→3’)** |
| --- | --- | --- |
| 1 | CUM1/F | GCTTTTCTGGATTCACCAACTGTGGCCGGCTGGGT |
| 2 | CUM1/R | ACCCAGCCGGCCACAGTTGGTGAATCCAGAAAAGC |
| 3 | CUM2/F | TGGGGTTCGAAATGACCAACCAAGCGACGCCCAAC |
| 4 | CUM2/R | GTTGGGCGTCGCTTGGTTGGTCATTTCGAACCCCA |
| 5 | CUM3/F | TCTGTCGATACCCCACCAAGACCCCATTGGGGCCA |
| 6 | CUM3/R | TGGCCCCAATGGGGTCTTGGTGGGGTATCGACAGA |
| 7 | pCRE/1 | TTCGAAATGACCTACCAAGCGACTTCGAAATGACCTAC |
| 8 | pCRE/2 | GTCGCTTGGTAGGTCATTTCGAAGTCGCTTGGTAGGT |
| 9 | pCRE/3 | TTCTTAATGACCGACCAAGCGACTTCTTAATGACCGAC |
| 10 | pCRE/4 | GTCGCTTGGTCGGTCATTAAGAAGTCGCTTGGTCGGT |
| 11 | Uchip/F | AAGAGTAGAGAAGAGTGTGGTAGTG |
| 12 | Uchip/R | GGGATATGATATGATGAAGTTTGTC |
| 13 | pyPro/F | CGGGATCCGATTACCTAAATAGCTTGGCGTAA |
| 14 | pyPro/R | CGGGATCCGGCGTAATCATGGTCATAGCTGTT |
| 15 | CaZF-RT/F | ATGGCTTTAGAGTTAGAAGCTTTCAATTCTTC |
| 16 | CaZF-RT/R | AGACGGATACAGTGTCGTTGAAGGCTGTGGATG |
| 17 | cpAct/F | TGGTTATTCTTTTACCACCTCAGCAG |
| 18 | cpAct/R | GGAACAGGACCTCTGGACATCT |
| 19 | NtAct/F | GACACTGCCAAGAGCAGCTC |
| 20 | NtAct/R | TGTTGCCATAGAGGTCCTTC |
| 21 | pgCAP2/F | GGAATTCCATATATGTTAGTGAAAAGCCATCATAA |
| 22 | pgCAP2/R | GGAATTCTCAAGACAATGAAGGCTGGGATGAAC |
