# Supplementary material for: Promoter of CaZF, a Chickpea Gene That Positively Regulates Growth and Stress Tolerance, Is Activated by an AP2-Family Transcription Factor CAP2
Source: PLoS One. 2013 Feb 13;8(2):e56737. doi: 10.1371/journal.pone.0056737 (PMC3572041; doi:10.1371/journal.pone.0056737)
Supplement: Table S2 — Functional categorization of ESTs generated by subtracted cDNA library between CaZF OX and vector control. (DOC) [file pone.0056737.s002.doc]

**Table S2.** Functional categorization of ESTs generated by subtracted cDNA library between *CaZF*OX and vector control.

| **Gene bank match** | | | | **Annotation** | | | **E-value** | **Acc. No.** | | |  |
| --- | --- | --- | --- | --- | --- | --- | --- | --- | --- | --- | --- |
| **Cell defense** | | | | | | |  |  | | |  |
| BAB09414.1 | | ABC transporter, ATP-binding protein-like [*Arabidopsis*] | | | | | 8E-56 | GO308234 | | |  |
| ABD28700.1 | | ATP binding , related [*Medicago truncatula*] | | | | | 1E-24 | GO308122 | | |  |
| CAH10191.1 | | Avirulence-like protein 1 [*Festuca arundinacea*] | | | | | 5E-62 | GO308065 | | |  |
| CAA06925.1 | | Avr9 elicitor response protein [*Nicotiana tabacum*] | | | | | 3E-62 | GO308172 | | |  |
| AAG43549.1 | | Avr9/Cf-9 rapidly elicited protein 1 | | | | | 7E-62 | GO308066 | | |  |
| AAG43549.1 | | Avr9/Cf-9 rapidly elicited protein 111B [*N. tabacum*] | | | | | 1E-41 | GO308198 | | |  |
| AAG43557.1 | | Avr9/Cf-9 rapidly elicited protein 65 [*N. tabacum*] | | | | | 2E-44 | GO308135 | | |  |
| AAB23374.1 | | Basic chitinase [*Nicotiana tabacum*] | | | | | 4E-86 | GO308216 | | |  |
| AAL26909.1 | | Dehydration-responsive protein RD22 [*Prunus persica*] | | | | | 1E-27 | GO308113 | | |  |
| ABB47791.2 | | Dehydration-responsive protein, putative, expr | | | | | 8E-45 | GO308112 | | |  |
| ACA24495.1 | | Gamma reponse I-like protein [*Cucumis sativus*] | | | | | 3E-66 | GO308034 | | |  |
| AAB48305.1 | | Hs1pro-1 [*Beta procumbens*] | | | | | 4E-77 | GO308211 | | |  |
| AAN17462.1 | | Hypersensitive-induced reaction protein 1 [*Hordeum vulgare*] | | | | | 2E-79 | GO308047 | | |  |
| AAT45202.1 | | Lipid transfer protein 1 precursor [*Nicotiana tabacum*] | | | | | 1E-55 | GO308050 | | |  |
| AAS13435.1 | | Lipid-transfer protein [*Nicotiana attenuata*] | | | | | 1E-47 | GO308200 | | |  |
| BAE98764.1 | | MRP-like ABC transporter [*Arabidopsis thaliana*] | | | | | 4E-71 | GO308103 | | |  |
| BAD07484.1 | | PDR-type ABC transporter 2 [*Nicotiana tabacum*] | | | | | 2E-88 | GO308105 | | |  |
| ABD33460.1 | | Putative NBS-LRR resistance disease protein rsp22 [*Ipomoea*] | | | | | 2E-45 | GO308159 | | |  |
| CAF33484.1 | | Putative pathogenesis-related protein [*Cucumis* | | | | | 5E-15 | GO308060 | | |  |
| ACG50004.1 | | Salt responsive protein 2 [*Solanum lycopersicum*] | | | | | 3E-88 | GO308149 | | |  |
| ABJ89813.1 | | Wound-induced protein kinase [*Nicotiana attenuata*] | | | | | 7E-65 | GO308130 | | |  |
| AAX20033.1 | | GDSL-lipase protein [*Capsicum annuum*] | | | | | 4E-87 | GO308055 | | |  |
| AAG34872.1 | | In2-1 protein [*Glycine max*] | | | | | 5E-62 | GO308169 | | |  |
| BAG68298.1 | | Serine palmitoyltransferase [*Nicotiana benthamiana*] | | | | | 2E-82 | GO308185 | | |  |
| ABE98329.1 | | SRC2-like protein [*Nicotiana benthamiana*] | | | | | 3E-5 | GO308136 | | |  |
| CAA47374.1 | | Prb-1b [*Nicotiana tabacum*] | | | | | 9E-65 | GO308054 | | |  |
| **Cellular organization** | | | | | | |  |  | | |  |
| CAD13178.1 | | Alpha-tubulin [*Nicotiana tabacum*] | | | | | 3E-64 | GO308121 | | |  |
| AAR37366.1 | | Beta-tubulin [*Nicotiana attenuata*] | | | | | 1E-151 | GO308028 | | |  |
| AAO03579.1 | | Cellulose synthase-like protein D4 [*Populus trichocarpa*] | | | | | 1E-141 | GO308219 | | |  |
| CAA18105.1 | | Glycine-rich protein [*Arabidopsis thaliana*] | | | | | 4E-5 | GO308131 | | |  |
| BAA83710.1 | | Heat shock factor [*Nicotiana tabacum*] | | | | | 3E-37 | GO308183 | | |  |
| AAR17080.1 | | Heat shock protein 70-3 [*Nicotiana tabacum*] | | | | | 3E-110 | GO308064 | | |  |
| ACD45076.1 | | Heat-shock protein 70 [*Dactylis glomerata*] | | | | | 3E-69 | GO308058 | | |  |
| CAB01913.1 | | Histone H4 homologue [*Sesbania rostrata*] | | | | | 7E-38 | GO308072 | | |  |
| AAR12194.1 | | Molecular chaperone Hsp90-2 [*Nicotiana benthamiana*] | | | | | 5E-118 | GO308096 | | |  |
| AAM65650.1 | | Pectinesterase, putative [*Arabidopsis thaliana*] | | | | | 2E-90 | GO308217 | | |  |
| CAB75430.1 | | Putative 16kda membrane protein [*Nicotiana tabacum*] | | | | | 3E-64 | GO308043 | | |  |
| CAI53895.2 | | Putative receptor associated protein [*Capsicum anuum*] | | | | | 2E-107 | GO308162 | | |  |
| AAC79095.1 | | Putative ribonucleoprotein [*Arabidopsis thaliana*] | | | | | 1E-69 | GO308046 | | |  |
| AAZ80876.1 | | Putative sorbitol transporter [*N. langsdorffii* x *N. sanderae*] | | | | | 2E-60 | GO308038 | | |  |
| AAS46241.1 | | Xyloglucan endotransglucosylase-hydrolase XTH3 [*L. esculentum*] | | | | | 4E-125 | GO308192 | | |  |
| AAS46244.1 | | Xyloglucan endotransglucosylase-hydrolase XTH9 [*L. esculentum*] | | | | | 3E-48 | GO308108 | | |  |
| AAG43444.1 | | Xyloglucan endotransglycosylase [*Solanum*] | | | | | 1E-118 | GO308107 | | |  |
| CAA58003.1 | | Xyloglucan endo-transglycosylase [*Solanum lycpersicum*] | | | | | 2E-14 | GO308093 | | |  |
| AAQ55288.2 | | Phytocalpain [*Nicotiana benthamiana*] | | | | | 1E-69 | GO308218 | | |  |
| **Energy metabolism** | | | | | | |  |  | | |  |
| CAA86468.1 | | 1-aminocyclopropane-1-carboxylate deaminase [*Nicotiana*] | | | | | 8E-104 | GO308070 | | |  |
| BAA07828.1 | | 4-coumarate:coenzyme A ligase [*Nicotiana tabacum*] | | | | | 9E-93 | GO308138 | | |  |
| CAA70968.2 | | Amino acid transporter [*Solanum tuberosum*] | | | | | 6E-89 | GO308030 | | |  |
| BAA25685.1 | | Arginine decarboxylase [*Nicotiana sylvestris*] | | | | | 4E-13 | GO308119 | | |  |
| AAK13318.1 | | ATP:citrate lyase [*Capsicum annuum*] | | | | | 2E-45 | GO308049 | | |  |
| CAA65063.1 | | c subunit of V-type ATPase [*Nicotiana tabacum*] | | | | | 4E-22 | GO308171 | | |  |
| AAA57551.1 | | Catalase | | | | | 7E-62 | GO308238 | | |  |
| AAF81310.1 | | Contains similarity to a dehydrogenase [*Arabidopsis thaliana*] | | | | | 9E-17 | GO308083 | | |  |
| AAT84461.1 | | Cytochrome b5 isoform Cb5-D [*Vernicia fordii*] | | | | | 9E-7 | GO308061 | | |  |
| BAA10929.1 | | Cytochrome P450 like_TBP [*Nicotiana tabacum*] | | | | | 1E-39 | GO308073 | | |  |
| AAB58728.1 | | Cytosolic NADP-malic enzyme [*Lycopersicon esculentum*] | | | | | 1E-39 | GO308062 | | |  |
| ABI98681.2 | | Cytosolic NADP-malic enzyme [*Nicotiana tabacum*] | | | | | 1E-82 | GO308068 | | |  |
| AAB02006.1 | | Epoxide hydrolase [*Nicotiana tabacum*] | | | | | 2E-100 | GO308227 | | |  |
| CAA54045.1 | | H(^+^)-transporting ATPase [*Solanum tuberosum*] | | | | | 3E-100 | GO308158 | | |  |
| ABC01898.1 | | Mitochondrial carrier-like protein [*Solanum tabacum*] | | | | | 2E-102 | GO308205 | | |  |
| ABR67418.1 | | Mitochondrial FAD carrier [*Cucumis melo*] | | | | | 1E-30 | GO308129 | | |  |
| BAB18781.1 | | Mitochondrial protein-like protein [*Cucumis melo*] | | | | | 9E-75 | GO308199 | | |  |
| CAC19856.1 | | Mitochondrial succinate dehydrogenase Fe^+2^-sulphur subunit [*A.thaliana*] | | | | | 1E-72 | GO308123 | | |  |
| CAA69601.2 | | NADH glutamate dehydrogenase [*Nicotiana*] | | | | | 5E-139 | GO308127 | | |  |
| CAA69601.2 | | NADH glutamate dehydrogenase [*Nicotiana*] | | | | | 4E-139 | GO308098 | | |  |
| CAD33241.1 | | Putative mitochondrial NAD-dependent malate dehydrogenase [*Solanum*] | | | | | 2E-126 | GO308228 | | |  |
| ACF17669.1 | | | Putative pyruvate dehydrogenase E1 alpha subunit | | | 5E-143 | | | GO308128 | | |
| AAC48918.1 | | Salicylic acid binding catalase | | | | | 2E-141 | GO308039 | | |  |
| AAD33072.1 | | Secretory peroxidase [*Nicotiana tabacum*] | | | | | 3E-126 | GO308032 | | |  |
| BAG80556.1 | | UDP-glucose:glucosyltransferase [*Lycium barbarum*] | | | | | 3E-40 | GO308053 | | |  |
| **Hormone biosynthesis** | | | | | | |  |  | | |  |
| AAP83138.1 | | Lipoxygenase [*Nicotiana attenuata*] | | | | | 2E-111 | GO308156 | | |  |
| ABY55855.1 | | S-adenosylmethionine decarboxylase 2 [*Solanum*] | | | | | 2E-44 | GO308120 | | |  |
| **Metabolism** | | | | | | |  |  | | |  |
| CAA45700.1 | | 23 kDa polypeptide of water-oxidizing complex of PS II [*N. tabacum*] | | | | | 1E-74 | GO308026 | | |  |
| BAD80839.1 | | 2-Hydroxyisoflavanone dehydratase | | | | | 1E-23 | GO308029 | | |  |
| ABW17197.1 | | Alanine aminotransferase 2 [*Glycine max*] | | | | | 7E-92 | GO308195 | | |  |
| AAM75140.1 | | Alkaline alpha galactosidase II [*Cucumis melo*] | | | | | 8E-93 | GO308091 | | |  |
| BAE98988.1 | | Beta-1,4-N-acetylglucosaminyltransferase like protein | | | | | 4E-91 | GO308174 | | |  |
| AAK30294.1 | | Beta-amylase [*Castanea crenata*] | | | | | 4E-58 | GO308150 | | |  |
| CAA84525.1 | | Chlorophyll a,b binding protein type I [*Solanum*] | | | | | 7E-89 | GO308036 | | |  |
| AAO62942.1 | | Chlorophyll a/b binding protein [*Nicotiana tabacum*] | | | | | 2E-93 | GO308201 | | |  |
| CAK24966.1 | | Chlorophyll a/b binding protein [*Solanum*] | | | | | 9E-65 | GO308057 | | |  |
| AAB61236.1 | | Chlorophyll a/b-binding protein [*Mesembryanthem*] | | | | | 4E-38 | GO308193 | | |  |
| ABB55370.1 | | Chlorophyll a-b binding protein 3C-like [*Solanum*] | | | | | 1E-118 | GO308104 | | |  |
| ABC59516.1 | | Chloroplast photosystem II 22 kDa component | | | | | 4E-64 | GO308180 | | |  |
| ACB05667.1 | | Chloroplast rubisco activase [*Capsicum annuum*] | | | | | 3E-43 | GO308080 | | |  |
| AAF19345.1 | | Diacylglycerol acylCoA acyltransferase [*Nicotiana*] | | | | | 6E-76 | GO308052 | | |  |
| BAB03027.1 | | Glutamine-fructose-6-phosphate transaminase 2 [*Arabidopsis thaliana*] | | | | | 2E-89 | GO308077 | | |  |
| CAC80374.1 | | Glyceraldehyde-3-phosphate dehydrogenase [*Capsicum annum*] | | | | | 3E-46 | GO308090 | | |  |
| ABY20971.1 | | Glyceraldehyde-3-phosphate dehydrogenase A subunit | | | | | 8E-66 | GO308075 | | |  |
| AAC33509.1 | | Glycolate oxidase [*Nicotiana tabacum*] | | | | | 1E-17 | GO308067 | | |  |
| BAA25393.1 | | Light harvesting chlorophyll a/b-binding protein | | | | | 8E-117 | GO308242 | | |  |
| BAA25395.1 | | Light harvesting chlorophyll a/b-binding protein [*Nicotiana tabacum*] | | | | | 2E-119 | GO308044 | | |  |
| CAC12826.1 | | Malate dehydrogenase [*Nicotiana tabacum*] | | | | | 2E-42 | GO308194 | | |  |
| CAH60894.1 | Malate dehydrogenase [*Solanum lycopersicum*] | | | | 5E-78 | | | GO308097 | |  | |
| [ABI95860.1](http://www.ncbi.nlm.nih.gov/entrez/query.fcgi?cmd=Retrieve&db=Protein&list_uids=115361539&dopt=GenPept&RID=K6TDDHSW01R&log$=prottop&blast_rank=1) | | Methionine synthase [*N. suaveolens*] | | | | | 6E-105 | GO308025 | | |  |
| ABC55421.1 | | Myo-inositol-1-phosphate synthase [*Glycine max*] | | | | | 8E-115 | GO308175 | | |  |
| BAG09382.1 | | Peroxisomal glycolate oxidase | | | | | 3E-112 | GO308081 | | |  |
| BAG09382.1 | | Peroxisomal glycolate oxidase [*Glycine max*] | | | | | 3E-114 | GO308031 | | |  |
| BAB89366.1 | | Phosphoenolpyruvate carboxylase [*Nicotiana tabacum*] | | | | | 2E-83 | GO308243 | | |  |
| CAJ19272.1 | | Plastocyanin precursor [*Solanum commersonii*] | | | | | 2E-48 | GO308222 | | |  |
| BAA04634.1 | | PSI-H precursor [*Nicotiana sylvestris*] | | | | | 3E-08 | GO308173 | | |  |
| AAK72885.1 | | Putative lipid acyl hydrolase [*Oryza sativa*] | | | | | 5E-57 | GO308146 | | |  |
| AAK26130.1 | | Putative thiamin biosynthesis protein [*Oryza sativa*] | | | | | 8E-119 | GO308115 | | |  |
| CAB59430.1 | | Quinolinate phosphoribosyltransferase [*Nicotiana tabacum*] | | | | | 9E-62 | GO308187 | | |  |
| AAA34116.1 | | Ribulose-1, 5-bisphosphate carboxylase small subunit | | | | | 2E-24 | GO308230 | | |  |
| AAM62603.1 | | Rubisco expression protein, putative [*Arabidopsis thaliana*] | | | | | 5E-9 | GO308085 | | |  |
| AAF24126.1 | | Soluble starch synthase [*Arabidopsis thaliana*] | | | | | 1E-22 | GO308178 | | |  |
| ABV25893.1 | | Starch synthase isoform I [*Manihot esculenta*] | | | | | 2E-22 | GO308186 | | |  |
| ABJ99591.1 | | Type III chlorophyll a/b-binding protein [*Lycopersicum*] | | | | | 8E-91 | GO308118 | | |  |
| AAB97152.1 | | Mg protoporphyrin IX chelatase [*Nicotiana tabacum*] | | | | | 9E-70 | GO308182 | | |  |
| ABY19385.1 | | Pheophorbide A oxygenase 2 [*Nicotiana tabacum*] | | | | | 2E-36 | GO308168 | | |  |
| **Protein metabolism** | | | | | | |  |  | | |  |
| AAF62403.1 | | Harpin inducing protein [*Nicotiana tabacum*] | | | | | 3E-38 | GO308206 | | |  |
| BAD07806.1 | | Putative HECT ubiquitin-protein ligase 3 [*Oryza sativa*] | | | | | 2E-74 | GO308240 | | |  |
| ACH87168.1 | | Senescence-related protein [*Camellia sinensis*] | | | | | 1E-56 | GO308224 | | |  |
| AAC00572.1 | | Similar to zinc metalloproteinases [*Arabidopsis thaliana*] | | | | | 1E-52 | GO308207 | | |  |
| ABD65144.1 | | Ubiquitin carboxyl-terminal hydrolase, putative [*Brassica oleracea*] | | | | | 2E-49 | GO308037 | | |  |
| **Signal transduction** | | | | | | |  |  | | |  |
| AAA86052.1 | | Abscisic stress ripening protein | | | | | 2E-26 | GO308190 | | |  |
| AAU14832.1 | | Adenosine kinase isoform 1S [*Nicotiana tabacum*] | | | | | 7E-44 | GO308059 | | |  |
| BAD73344.1 | | Auxin efflux carrier family protein-like [*Oryza sativa*] | | | | | 9E-12 | GO308100 | | |  |
| ABK41009.1 | | Auxin/indole-3-acetic acid [*Solanum tuberosum*] | | | | | 4E-31 | GO308155 | | |  |
| ABK06394.1 | | Ca2+-binding protein [*Citrus sinensis*] | | | | | 6E-29 | GO308099 | | |  |
| CAC43238.1 | | Calcium binding protein [*Sesbania rostrata*] | | | | | 6E-39 | GO308076 | | |  |
| AAF31152.1 | | Calcium-binding protein [*Olea europaea*] | | | | | 6E-22 | GO308163 | | |  |
| AAR99412.1 | | Calmodulin [*Arachis hypogaea*] | | | | | 1E-48 | GO308164 | | |  |
| AAQ63461.1 | | Calmodulin 4 [*Daucus carota*] | | | | | 2E-79 | GO308063 | | |  |
| ABM55247.1 | | Calmodulin-binding protein [*Beta vulgaris*] | | | | | 5E-79 | GO308134 | | |  |
| BAA95793.1 | | Carbonic anhydrase [*Nicotiana tabacum*] | | | | | 9E-54 | GO308221 | | |  |
| ABJ91223.1 | | CBL-interacting protein kinase 16 [*Populus trichocarpa*] | | | | | 5E-79 | GO308212 | | |  |
| AAF33670.1 | | Cyclic nucleotide-gated calmodulin-binding ion channel [*N. tabacum*] | | | | | 1E-110 | GO308125 | | |  |
| BAD00043.1 | | MAP kinase phosphatase [*Nicotiana tabacum*] | | | | | 2E-71 | GO308232 | | |  |
| BAC57589.1 | | Membrane located receptor-like protein [*Nicotiana tabacum*] | | | | | 3E-06 | GO308191 | | |  |
| ABY85198.2 | | Mitogen activated protein kinase 1 [*Datura metel*] | | | | | 2E-15 | GO308229 | | |  |
| CAA58594.1 | | Petunia Shaggy kinase 4 [Petunia x hybrida] | | | | | 1E-70 | GO308109 | | |  |
| CAA52979.1 | | Phosphate translocator [*Nicotiana tabacum*] | | | | | 4E-137 | GO308086 | | |  |
| ABP57375.1 | | Phosphoinositide-specific phospholipase C [*N. tabacum*]. | | | | | 4E-77 | GO308027 | | |  |
| BAD95059.1 | | Potassium transport protein-like [*Arabidopsis thaliana*] | | | | | 3E-59 | GO308048 | | |  |
| AAM63486.1 | | Protein kinase-like protein [*Arabidopsis thaliana*] | | | | | 6E-36 | GO308071 | | |  |
| AAQ67229.1 | | Protein phosphatase 2A catalytic subunit [*Nicotiana tabacum*] | | | | | 9E-55 | GO308196 | | |  |
| AAD11598.1 | | Putative calcium channel [*Arabidopsis thaliana*] | | | | | 9E-93 | GO308110 | | |  |
| CAC37356.1 | | Putative membrane protein [*Solanum tuberosum*] | | | | | 3E-35 | GO308235 | | |  |
| AAC33204.1 | | Putative protein kinase [*Arabidopsis thaliana*] | | | | | 1E-30 | GO308210 | | |  |
| BAE99831.1 | | Putative receptor-like protein kinase [*Arabidopsis thaliana*] | | | | | 1E-85 | GO308116 | | |  |
| BAF02199.1 | | Putative serine/threonine protein kinase [*Arabidopsis thaliana*] | | | | | 9E-97 | GO308040 | | |  |
| BAD09759.1 | | Putative signal recognition particle receptor | | | | | 2E-76 | GO308239 | | |  |
| AAM44081.1 | | Type IIB calcium ATPase MCA5 [*Medicago truncatulata*] | | | | | 5E-81 | GO308151 | | |  |
| ABC01915.1 | | Vacuolar sorting receptor protein PV72-like protein | | | | | 2E-62 | GO308142 | | |  |
| ABD32569.1 | | V-ATPase subunit C [*Medicago truncatula*] | | | | | 4E-111 | GO308203 | | |  |
| ABG66292.1 | | VHS domain-containing protein, putative, expressed [*Oryza sativa*] | | | | | 5E-33 | GO308231 | | |  |
| **Transcription** | |  | | | | |  |  | | |  |
| CAB45908.1 | | Beta-COP-like protein [*Arabidopsis thaliana*] | | | | | 3E-78 | GO308244 | | |  |
| AAO13360.1 | | DREB3 [*Lycopersicon esculentum*] | | | | | 1E-31 | GO308209 | | |  |
| ACE73695.1 | | DREB3 [*Nicotiana tabacum*] | | | | | 1E-86 | GO308139 | | |  |
| ACE73696.1 | | DREB4 [*Nicotiana tabacum*] | | | | | 7E-110 | GO308056 | | |  |
| ABV89652.1 | | Early-responsive to dehydration 4 [*Brassica rapa*] | | | | | 8E-94 | GO308189 | | |  |
| AAX20034.1 | | Ethylene responsive element binding protein C1 [*Capsicum anuum*] | | | | | 9E-25 | GO308226 | | |  |
| AAG51287.1 | | Helicase, putative [*Arabidopsis thaliana*] | | | | | 7E-46 | GO308153 | | |  |
| CAF74711.1 | | MYC transcription factor [*Solanum tuberosum*] | | | | | 7E-30 | GO308106 | | |  |
| AAF66823.1 | | Poly(A)-binding protein [*Nicotiana tabacum*] | | | | | 2E-149 | GO308111 | | |  |
| AAL87345.1 | | Putative chloroplast nucleoid DNA-binding protein | | | | | 7E-38 | GO308167 | | |  |
| CAD59768.1 | | Putative reverse transcriptase [*Cicer arietinum*] | | | | | 8E-12 | GO308095 | | |  |
| BAD10335.1 | | Putative transcriptional regulator [*Oryza sativa*] | | | | | 2E-43 | GO308042 | | |  |
| ACF74549.1 | | RAV transcription factor [*Nicotiana tabacum*] | | | | | 2E-62 | GO308051 | | |  |
| CAB10245.1 | | RNA polymerase II fifth largest subunit like protein [*A. thaliana*] | | | | | 6E-62 | GO308082 | | |  |
| BAA77383.1 | | Transcription factor NtWRKY2 [*Nicotiana tabacum*] | | | | | 7E-137 | GO308041 | | |  |
| BAF48804.1 | | Wound-responsive AP2 like factor 2 [*Nicotiana tabacum*] | | | | | 2E-72 | GO308088 | | |  |
| ACF04195.1 | | WRKY [*Solanum lycopersicum*] | | | | | 4E-12 | GO308147 | | |  |
| BAB61055.1 | | WRKY DNA-binding protein [*Nicotiana tabacum*] | | | | | 1E-137 | GO308154 | | |  |
| ACJ04728.1 | | WRKY transcription factor-30 [*Capsicum annuum*] | | | | | 1E-37 | GO308213 | | |  |
| AAX95671.1 | | Zn-finger in Ran binding protein and others, putative [*Oryza sativa*] | | | | | 4E-18 | GO308045 | | |  |
| ABE01085.1 | | BTF3 [*Nicotiana benthamiana*] | | | | | 5E-54 | GO308177 | | |  |
| ACD49740.1 | | BURP domain-containing protein [*Solanum lycopersicum*] | | | | | 2E-55 | GO308181 | | |  |
| ABE02823.1 | | GRAS1 [*Nicotiana tabacum*] | | | | | 6E-114 | GO308233 | | |  |
| ABD72959.1 | | GRAS2 [*Solanum lycopersicum*] | | | | | 1E-112 | GO308157 | | |  |
| AAM47025.1 | | Nam-like protein 1 [*Petunia x hybrida*] | | | | | 1E-65 | GO308102 | | |  |
| AAM34773.1 | | Nam-like protein 10 [*Petunia x hybrida*] | | | | | 1E-59 | GO308141 | | |  |
| AAM34770.1 | | Nam-like protein 7 [*Petunia x hybrida*] | | | | | 3E-88 | GO308170 | | |  |
| BAA33810.1 | | Phi-1 [*Nicotiana tabacum*] | | | | | 7E-140 | GO308069 | | |  |
| CAF18246.1 | | STY-L protein [*Antirrhinum majus*] | | | | | 2E-104 | GO308245 | | |  |
| CAA88492.1 | | TAF-2 [*Nicotiana tabacum*] | | | | | 2E-76 | GO308140 | | |  |
| BAA87058.1 | | WIZZ [*Nicotiana tabacum*] | | | | | 4E-42 | GO308236 | | |  |
| **Translation** | |  | | | | |  |  | | |  |
| AAM63791.1 | | 40S ribosomal protein S19-like [*Arabidopsis thaliana*] | | | | | 8E-10 | GO308223 | | |  |
| CAA09042.1 | | 40S ribosomal protein S6 [*Cicer arietinum]* | | | | | 3E-43 | GO308133 | | |  |
| ABA40437.1 | | 40S ribosomal protein S7-like protein [Solanum | | | | | 1E-67 | GO308084 | | |  |
| ABR25618.1 | | 60S ribosomal protein l2 [*Oryza sativa* (*indica*)] | | | | | 3E-21 | GO308035 | | |  |
| ABA40469.1 | | 60S ribosomal protein L21-like protein [*Solanum*] | | | | | 4E-39 | GO308184 | | |  |
| ABA40469.1 | | 60S ribosomal protein L21-like protein | | | | | 4E-49 | GO308074 | | |  |
| ABX71676.1 | | 60S ribosomal protein L6-like protein | | | | | 2E-25 | GO308144 | | |  |
| BAA09709.1 | | Elongation factor-1 alpha [*Nicotiana tabacum*] | | | | | 7E-09 | GO308225 | | |  |
| CAB10520.1 | | Ribosomal protein [*Arabidopsis thaliana*] | | | | | 2E-22 | GO308214 | | |  |
| ABB72816.1 | | Ribosomal protein L24-like protein [*Solanum* ] | | | | | 5E-28 | GO308237 | | |  |
| **Unclassified** | |  | | | | |  |  | | |  |
| CAD29735.1 | | Allene oxide synthase [*Solanum tuberosum*] | | | | | 7E-107 | GO308148 | | |  |
| AAW02789.1 | | Aluminum-induced protein [*Codonopsis lanceolata*] | | | | | 2E-36 | GO308152 | | |  |
| AAM98103.1 | | At1g02660/T14P4_9 [*Arabidopsis thaliana*] | | | | | 2E-20 | GO308092 | | |  |
| AAL24253.1 | | AT4g20170/F1C12_90 [*Arabidopsis thaliana*] | | | | | 1E-57 | GO308176 | | |  |
| AAO42869.1 | | At5g42860 [*Arabidopsis thaliana*] | | | | | 4E-15 | GO308126 | | |  |
| BAD88358.1 | | CBS domain containing protein-like [*Oryza sativa*] | | | | | 8E-34 | GO308137 | | |  |
| AAK69757.1 | | Chromomethylase CMT2 [*Arabidopsis thaliana*] | | | | | 1E-82 | GO308220 | | |  |
| ABX09988.1 | | Cullin 4 [*Solanum lycopersicum*] | | | | | 3E-113 | GO308166 | | |  |
| EEB19994.1 | | Cylicin-1, putative [*Pediculus humanus* corporis] | | | | | 5.7 | GO308078 | | |  |
| CAC05495.1 | | DNA gyrase subunit B-like protein [*Arabidopsis thaliana*] | | | | | 4E-115 | GO308087 | | |  |
| AAG00249.1 | | F1N21.14 [*Arabidopsis thaliana*] | | | | | 1E-83 | GO308160 | | |  |
| AAG00256.1 | | F1N21.7 [*Arabidopsis thaliana*] | | | | | 3E-32 | GO308114 | | |  |
| CAD37200.1 | | GDA2 protein [*Pisum sativum*] | | | | | 1E-50 | GO308132 | | |  |
| ACF74342.1 | | Gonadotropin beta chain [*Arachis hypogaea*] | | | | | 6E-15 | GO308188 | | |  |
| CAB61744.1 | | Hypothetical protein [*Cicer arietinum*] | | | | | 5E-34 | GO308145 | | |  |
| CAI84656.1 | | Hypothetical protein [*Nicotiana tabacum*] | | | | | 8E-08 | GO308208 | | |  |
| CAN76368.1 | | Hypothetical protein [*Vitis vinifera*] | | | | | 3E-83 | GO308124 | | |  |
| EEA59336.1 | | Hypothetical protein BRAFLDRAFT_83344 [Branchi | | | | | 3.8 | GO308079 | | |  |
| EAZ28165.1 | | Hypothetical protein OsJ_011648 [*Oryza sativa*] | | | | | 1E-17 | GO308204 | | |  |
| ABA46779.1 | | Meloidogyne-induced giant cell protein-like protein [*Solanum*] | | | | | 6E-43 | GO308143 | | |  |
| BAA06151.1 | | Pit2 [*Nicotiana tabacum*] | | | | | 7E-44 | GO308161 | | |  |
| CAB79691.1 | | Putative protein [*Arabidopsis thaliana*] | | | | | 1E-52 | GO308246 | | |  |
| BAD45511.1 | | Putative RAD26 [*Oryza sativa* Japonica Group] | | | | | 2E-5 | GO308033 | | |  |
| BAD45605.1 | | Putative t-complex protein 1 theta chain [*Oryza sativa*] | | | | | 3E-103 | GO308101 | | |  |
| AAK11255.1 | | Regulator of gene silencing [*Nicotiana tabacum*] | | | | | 6E-75 | GO308197 | | |  |
| BAF47120.1 | | Translationally controlled tumor protein like protein [*Nicotiana tabacum*] | | | | | 1E-43 | GO308179 | | |  |
| ABA59556.1 | | U-box protein [*Capsicum annuum*] | | | | | 2E-63 | GO308117 | | |  |
| ABK92936.1 | | Unknown [*Populus trichocarpa*] | | | | | 8E-119 | GO308165 | | |  |
| ABB87113.1 | | Unknown [*Solanum tuberosum*] | | | | | 2E-62 | GO308241 | | |  |
| ACG30009.1 | | Unknown [*Zea mays*] | | | | | 2E-78 | GO308089 | | |  |
| BAD43828.1 | | Unknown protein [*Arabidopsis thaliana*] | | | | | 5E-13 | GO308215 | | |  |
| BAA89236.1 | | Unnamed protein product [*Nicotiana tabacum*] | | | | | 8E-70 | GO308094 | | |  |
| CAO45813.1 | | Unnamed protein product [*Vitis vinifera*] | | | | | 4E-122 | GO308202 | | |  |
