# Supplementary figures and images for: Promoter of CaZF, a Chickpea Gene That Positively Regulates Growth and Stress Tolerance, Is Activated by an AP2-Family Transcription Factor CAP2
Source: PLoS One. 2013 Feb 13;8(2):e56737. doi: 10.1371/journal.pone.0056737 (PMC3572041; doi:10.1371/journal.pone.0056737)

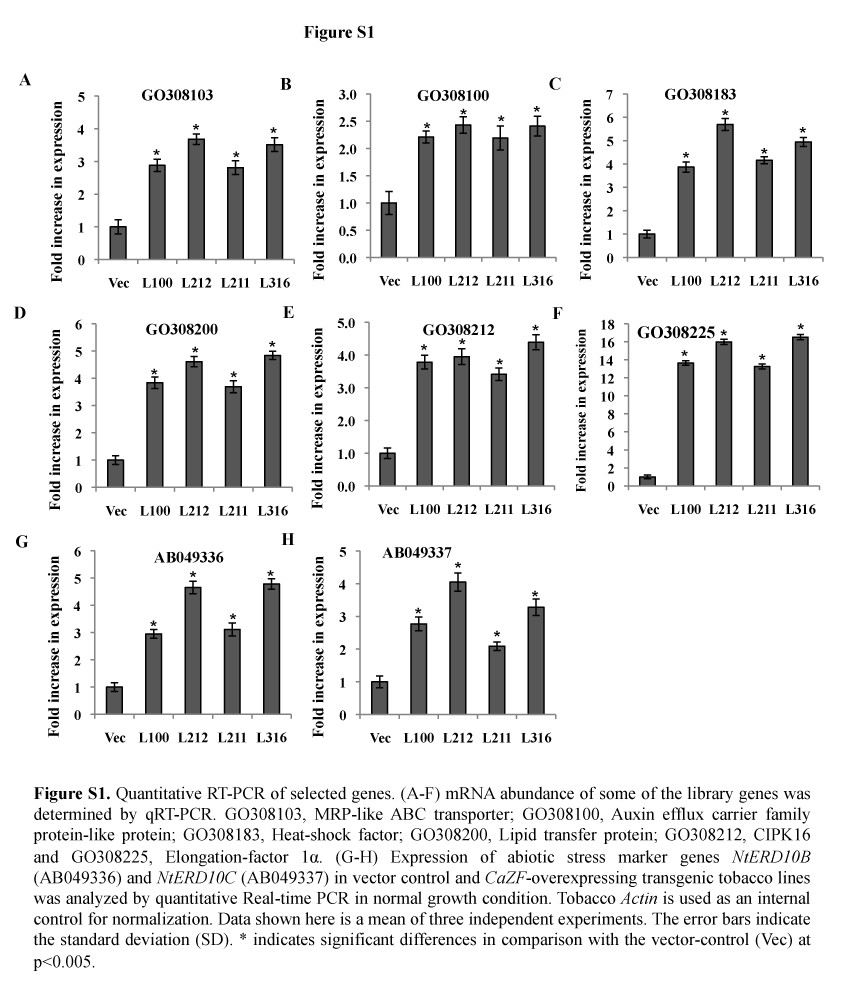

Supplement: Figure S1 — Quantitative RT-PCR of selected genes. (A–F) mRNA abundance of some of the library genes was determined by qRT-PCR. GO308103, MRP-like ABC transporter; GO308100, Auxin efflux carrier family protein-like protein; GO308183, Heat-shock factor; GO308200, Lipid transfer protein; GO308212, CIPK16 and GO308225, Elongation-factor 1α. (G–H) Expression of abiotic stress marker genes NtERD10B (AB049336) and NtERD10C (AB049337) in vector control and CaZF-overexpressing transgenic tobacco lines was analyzed by quantitative Real-time PCR in normal growth condition. Tobacco Actin is used as an internal control for normalization. Data shown here is a mean of three independent experiments. The error bars indicate the standard deviation (SD). * indicates significant differences in comparison with the vector-control (Vec) at p<0.005. (TIF) [file pone.0056737.s003.tif]

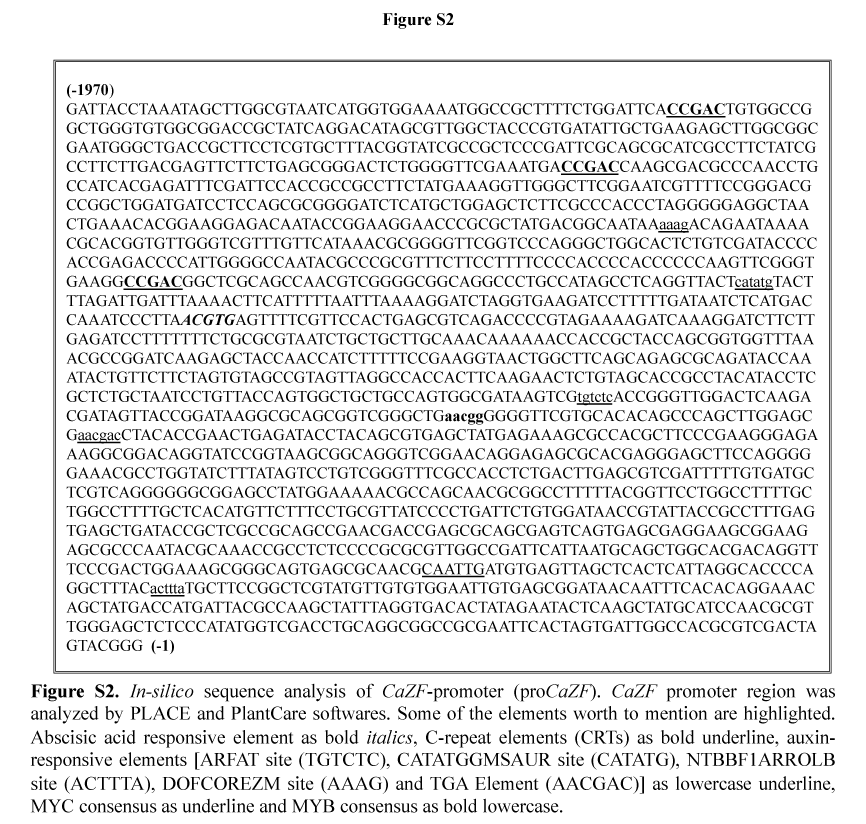

Supplement: Figure S2 — In-silico sequence analysis of CaZF -promoter (pro CaZF ). CaZF promoter region was analyzed by PLACE and PlantCare softwares. Some of the elements worth to mention are highlighted. Abscisic acid responsive element as bold italics, C-repeat elements (CRTs) as bold underline, auxin-responsive elements [ARFAT site (TGTCTC), CATATGGMSAUR site (CATATG), NTBBF1ARROLB site (ACTTTA), DOFCOREZM site (AAAG) and TGA Element (AACGAC)] as lowercase underline, MYC consensus as underline and MYB consensus as bold lowercase. (TIF) [file pone.0056737.s004.tif]

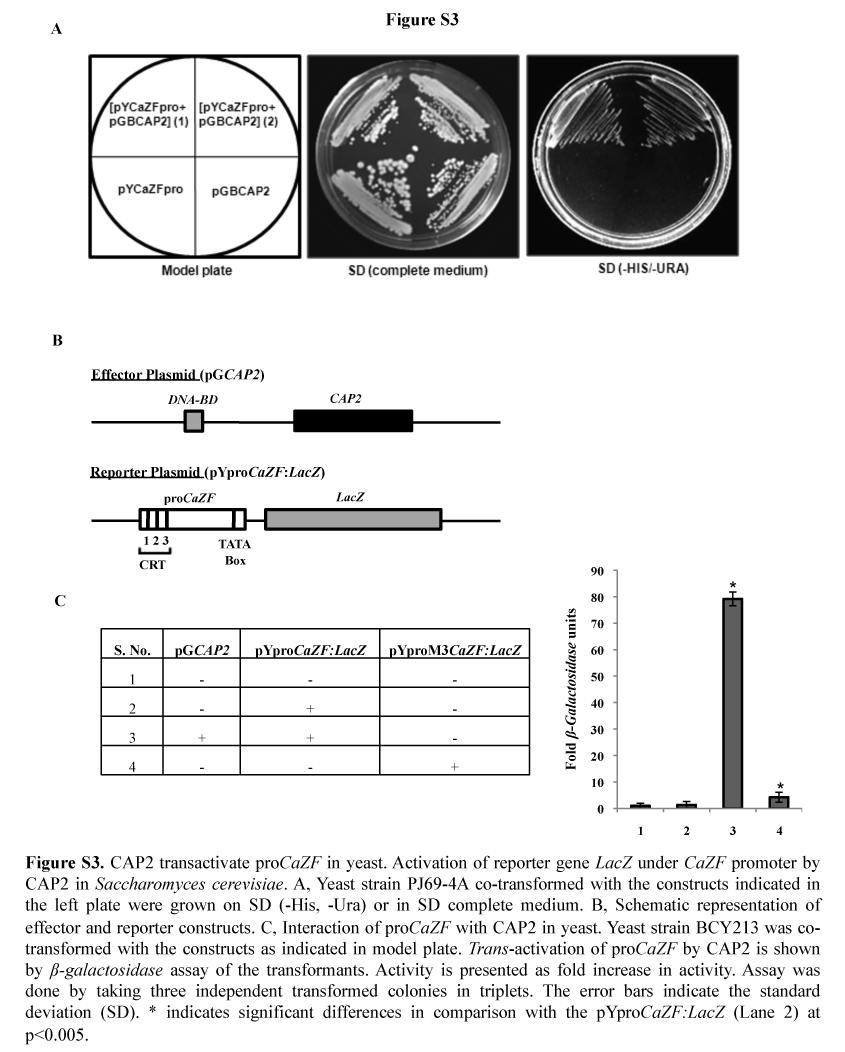

Supplement: Figure S3 — CAP2 transactivate pro CaZF in yeast. Activation of reporter gene LacZ under CaZF promoter by CAP2 in Saccharomyces cerevisiae. Activation of reporter gene LacZ under CaZF promoter by CAP2 in Saccharomyces cerevisiae. A, Yeast strain PJ69-4A co-transformed with the constructs indicated in the left plate were grown on SD (-His, -Ura) or in SD complete medium. B, Schematic representation of effector and reporter constructs. C, Interaction of proCaZF with CAP2 in yeast. Yeast strain BCY213 was co-transformed with the constructs as indicated in model plate. Trans-activation of proCaZF by CAP2 is shown by β-galactosidase assay of the transformants. Activity is presented as fold increase in activity. Assay was done by taking three independent transformed colonies in triplets. The error bars indicate the standard deviation (SD). * indicates significant differences in comparison with the pYproCaZF:LacZ (Lane 2) at p<0.005. (TIF) [file pone.0056737.s005.tif]

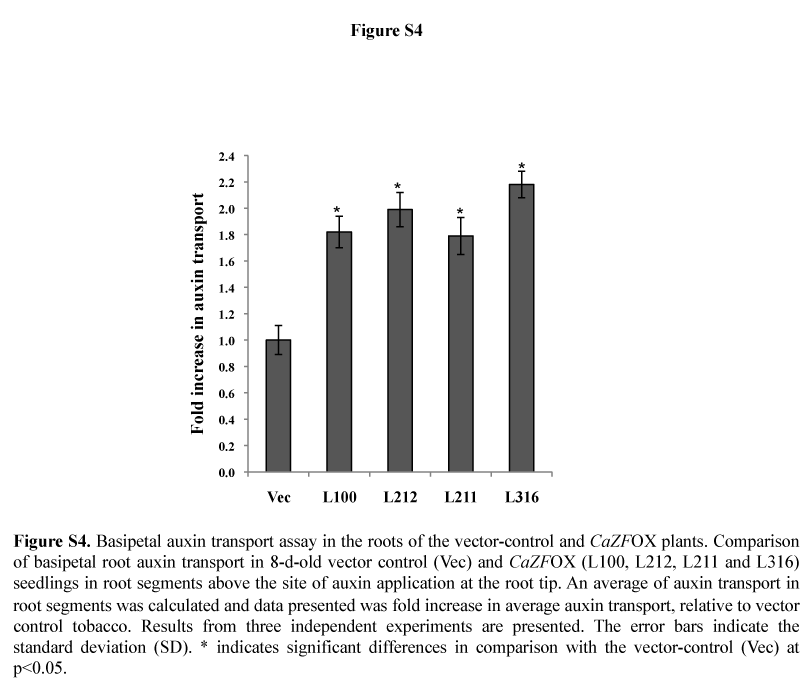

Supplement: Figure S4 — Basipetal-auxin transport assay in the roots of the vector-control and CaZF OX plants. Comparison of basipetal root auxin transport in 8-d-old vector control (Vec) and CaZFOX (L100, L212, L211 and L316) seedlings in root segments above the site of auxin application at the root tip. An average of auxin transport in root segments was calculated and data presented was fold increase in average auxin transport, relative to vector control tobacco. Results from three independent experiments are presented. The error bars indicate the standard deviation (SD). * indicates significant differences in comparison with the vector-control (Vec) at p<0.05. (TIF) [file pone.0056737.s006.tif]
