# Supplementary material for: Promoter of CaZF, a Chickpea Gene That Positively Regulates Growth and Stress Tolerance, Is Activated by an AP2-Family Transcription Factor CAP2
Source: PLoS One. 2013 Feb 13;8(2):e56737. doi: 10.1371/journal.pone.0056737 (PMC3572041; doi:10.1371/journal.pone.0056737)
Supplement: Methods S1 — Protoplast isolation and co-transfection analysis, Chromatin immunoprecipitation (ChIP) and Auxin transport assay. (DOC) [file pone.0056737.s007.doc]

## Methods S1.

## Protoplast isolation and co-transfection analysis

## Briefly, BY-2 cell suspension cultures were grown in BY-2 culture medium (MS salts, 1 mg/l thiamine-HCl, 370 mg/l KH_2_PO_4_, 30 g/l sucrose and 2 mg/l 2,4-D, pH 5.8) at 28^0^C, 140 rpm . 50 ml of suspension culture was pelleted at 1000 rpm at RT for 5 min. The pellet was gently re-suspended in 50 ml of protoplast enzyme digestion solution (protoplast isolation solution, PIS supplemented with 1 % cellulose R10, 0.5 % macerozyme and 0.1 % pectolyase Y23, pH-5.7, filter sterilized) and incubated at 28^0^C, 60 rpm for 1-2 h. Digested suspension culture was pelleted at 1000 rpm, RT for 5 min. The protoplasts were washed twice with PIS (7.4 g/l CaCl_2_.2H_2_O, 1 g/l NaOAc, and 45 g/l mannitol). The pellet was re-suspended in 5 ml of PIS, loaded onto 50 ml of floating solution (99 mg/l myo-inositol, 2.88 g/l L-proline, 100 mg/l enzymatic casein hydrolysate, 102.6 g/l sucrose, 97.6 mg/l MES buffer, 4.3 g/l MS salts, 1 mg/l vitamin B1, 370 mg/l KH_2_PO_4_, pH 5.7, filter sterilized) and pelleted at 1000 rpm, 5 min at RT. Floating protoplasts were gently removed, washed twice with PIS, and suspended in 10 ml W5 solution (154 mM NaCl, 125 mM CaCl_2_, 5 mM KCl, 2 mM MES, pH 5.7, filter sterilized). Proplasts were centrifuged at 1000 rpm, RT for 5 min, resuspended again in 10 ml of W5 solution and incubated for 30 min on ice. The protoplasts were again pelleted at 1000 rpm, RT for 5 min and suspended in MMg solution (0.6 M mannitol, 15 mM MgCl_2_, 4 mM MES, pH 5.7, filter sterilized) at a density of 1×10^6^ cells/ml. ~10 μg DNA (Control plasmids with or without proCaZF:GUS/CaMV35S:c-Myc-CAP2 plasmids, as shown in figure) was mixed with 100 μl protoplasts, followed by addition of 110 μl PEG solution (per ml solution; 0.4 g PEG 4000, 0.6 ml 1 M mannitol, 100 μl 1 M CaCl_2_), and incubated at RT for 30 min. After incubation 1 ml of W5 solution was added, centrifuged at 1000 rpm, RT for 5 min. The protoplast pellet was re-suspended in 1 ml of incubation solution (BY-2 culture medium with 0.6 M mannitol) and incubated in dark at 28^0^C for 48 h. In addition, 5 μg of p35S::EYFP1 plasmid was included in each transfection experiment as a control for normalizing transfection efficiency.

## Chromatin immunoprecipitation (ChIP)

## Tobacco leaf explants were transformed with proCaZF:GUS and CaMV-35S:c-Myc-CAP2 constructs along with control plasmids. 1.5 gm antibiotic-selected shoot lets were harvested and fixed with 1 % formaldehyde in fixation buffer (0.4 M sucrose, 10 mM Tris pH 8, 1 mM EDTA, 1 mM PMSF, 0.05 % Triton X-100) by vacuum infiltration at 28^0^C for 15 min. Formaldehyde was quenched by adding glycine to a final concentration of 125 mM and vaccum was applied for further 10 min. Tissue was washed repeatedly with ice-cold Tris buffered saline (TBS), dried and frozen in liquid nitrogen, and stored at -80^0^C until further use. Fixed cells were ground to a fine powder under liquid nitrogen and resuspended in 1 ml lysis buffer I (50 mM HEPES-KOH pH 7.5, 150 mM NaCl, 1 mM EDTA, 1 % Triton X-100, 0.1 % sodium-deoxycholate, 0.1 % SDS, 10 mM sodium-butyrate) and protease inhibitors [1 mM phenylmethylsulfonylflouoride (PMSF), 1 µg/ml each of aprotinin, leupeptin]. Cells were incubated on ice for 10 min and then sheared by repetitive sonication with a Vibracell sonifier (Sonics and Materials, USA) equipped with a microtip in an ice bath. The sheared chromatin was pelleted at 13 Krpm, 4^0^C for 15 min. The supernatant was incubated overnight with preimmune serum or anti-Myc antibody and 40 µl (40 % slurry) Protein A-sepharose beads (Amersham Biosciences, UK) at 4^0^C with gentle shaking. Beads were retained at 2 Krpm, 4^0^C for 2 min and washed twice each with wash buffer (150 mM NaCl, 0.1 % SDS, 0.5 % Triton-X-100, 1 mM EDTA and 20 mM HEPES, pH 8), LNDET buffer (0.25 M LiCl, 1 % NP-40, 1 % sodium deoxycholate, 1 mM EDTA and 10 mM Tris-HCl, pH 8) and TE (10 mM Tris-HCl, pH 8 and 1 mM EDTA). The chromatin-immune complex was eluted twice with 250 µl of elution buffer (1 % SDS and 0.1 M NaHCO_3_) at 65^0^C for 30 min. The DNA cross-linking was reversed by adding 5 M NaCl to the final concentration of 0.3 M at 65^0^C for overnight. Protein was degraded by adding protease solution (20 µg Proteinase-K, 10 mM EDTA and 40 mM Tris-HCl, pH 8) at 45^0^C for 2 h. Residual proteins were extracted with equal volume of phenol:chloroform:IAA (25:24:1) followed by ethanol precipitation of DNA. Immunoprecipitated DNA was amplified with promoter specific primers (Uchip/F and Uchip/R). In each PCR reaction, corresponding input was taken in parallel for PCR validation.

Auxin transport assay

Eight-day-old vertically grown seedling were used for the experiment. Auxin transport was measured essentially as previously described . Briefly, agar blocks of 2mm in diameter containing 7.7×10^−8^ M ^3^H-IAA (Amersham Biosciences) were placed at root tips in three independent experiments. After incubation for 3 h, a 2 mm section of the root above the agar block was dissected and discarded to avoid contamination from the agar block. Two consecutive 2-mm long segments above the incision line were then collected separately from fifteen roots. Pooled segments were placed into glass scintillation vials containing 5ml scintillation fluid. Radio-activities in these two pools of root segments were measured using a Beckman Coulter LS6500 Scintillation counter (Fullerton, CA, USA). The amount of the auxin transport was the average of three separate experiments with standard deviation.
